# *Tsc2* coordinates neuroprogenitor differentiation

**DOI:** 10.1016/j.isci.2023.108442

**Published:** 2023-11-14

**Authors:** Victoria A. Riley, Vijay Shankar, Jennie C. Holmberg, Aidan M. Sokolov, Victoria N. Neckles, Kaitlyn Williams, Rachel Lyman, Trudy F.C. Mackay, David M. Feliciano

**Affiliations:** 1 Department of Biological Sciences, Clemson University, Clemson, SC, USA; 2 Department of Biochemistry and Genetics, Clemson University, Clemson, SC, USA; 3 Center for Human Genetics, Clemson University, Greenwood, SC, USA; 4 Clemson University Genomics and Bioinformatics Facility (CUGBF), Clemson University, Clemson, SC, USA

**Keywords:** Molecular biology, Molecular mechanism of gene regulation, Neuroscience, Cell biology, Stem cells research, Transcriptomics

## Abstract

Neural stem cells (NSCs) of the ventricular-subventricular zone (V-SVZ) generate numerous cell types. The uncoupling of mRNA transcript availability and translation occurs during the progression from stem to differentiated states. The mTORC1 kinase pathway acutely controls proteins that regulate mRNA translation. Inhibiting mTORC1 during differentiation is hypothesized to be critical for brain development since somatic mutations of mTORC1 regulators perturb brain architecture. Inactivating mutations of *TSC1* or *TSC2* genes cause tuberous sclerosis complex (TSC). TSC patients have growths near the striatum and ventricles. Here, it is demonstrated that V-SVZ NSC *Tsc2* inactivation causes striatal hamartomas. *Tsc2* removal altered translation factors, translatomes, and translational efficiency. Single nuclei RNA sequencing following *in vivo* loss of *Tsc2* revealed changes in NSC activation states. The inability to decouple mRNA transcript availability and translation delayed differentiation leading to the retention of immature phenotypes in hamartomas. Taken together, *Tsc2* is required for translational repression and differentiation.

## Highlights

• Tsc2 mutant neural stem cells generate hamartomas containing ectopic neurons
• Tsc2 mutant neural stem cells have elevated mTORC1 activity and altered translation
• Tsc2 mutant neural stem cells have altered transitional states
• Tsc2 mutant glia retain stem-like phenotypes

## Graphical abstract

**Figure:**
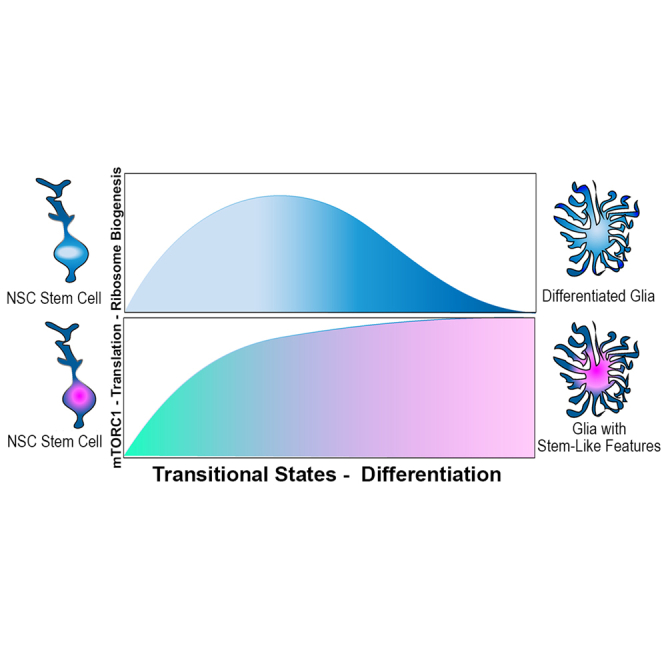

## Introduction

Embryonic neural stem cells (NSCs) that reside along the walls of the lateral ventricles of the brain form a stratified region called the ventricular-subventricular zone (V-SVZ).^1^ NSCs of dorsal, lateral, ventral, and caudal portions of the V-SVZ generate distinct cell types.^2^ The capacity of NSCs to generate these unique cell types is determined in part by genetically encoded transcription factors that specify programs of mRNA transcription.^3^^,^^4^^,^^5^^,^^6^^,^^7^ Extracellular factors influence transcriptional programs and cellular identity, however, the role of post-transcriptional processes regulating which mRNAs are translated into functional proteins is less clear.^8^ As one might expect, gene mutations that alter mRNA translation in NSCs and their progeny underlie a range of neurodevelopmental disorders characterized by cytoarchitectural aberrations associated with neurological manifestations.^9^^,^^10^^,^^11^

Tuberous sclerosis complex (TSC) is a genetic disorder affecting approximately 1 million patients worldwide.^12^ TSC is caused by inactivating mutations in *TSC1* or *TSC2*.^13^^,^^14^ The proteins encoded by *TSC1* and *TSC2* inhibit the translation regulatory protein mTOR.^15^^,^^16^^,^^17^^,^^18^ TSC patients have numerous types of growths called hamartomas and malformations including in the brain.^19^ Neurological manifestations in TSC are the greatest cause of morbidity in patients and includes seizures, intellectual disability, and a range of TSC associated neuropsychiatric disorders (TANDs) such as behavioral, psychiatric, neuropsychological, and autistic symptoms.^12^^,^^20^^,^^21^^,^^22^ Malformations within the cerebral cortex called cortical tubers resemble focal cortical dysplasia (II) and are epileptogenic foci that are a leading cause of patient seizures.^23^^,^^24^^,^^25^^,^^26^^,^^27^^,^^28^^,^^29^ As one would expect, surgical resection of epileptogenic tubers reduces seizure burden.^23^^,^^25^^,^^30^ Tubers can be detected during late gestation and are therefore thought to be a byproduct of abnormal development. Tubers are comprised of mislaminated cortical regions having cytomegalic excitatory neurons with hyperactive mTOR.^31^ Radial glia are embryonic NSCs that generate excitatory cortical neurons.^32^^,^^33^ Loss of TSC genes in radial glia is the most parsimonious explanation for how tubers form. In support of this hypothesis, removal of TSC genes from radial glia or excitatory neurons in mice recapitulates most phenotypes including cortical hyperexcitability.^34^^,^^35^^,^^36^^,^^37^^,^^38^^,^^39^^,^^40^^,^^41^ In contrast, loss of *Tsc1* from inhibitory neurons does not cause seizures.^42^^,^^43^

The cause of TANDs are less clear. A possible culprit is the additional hamartomatous growths and smaller microlesions found within the brain of TSC patients.^44^ Subependymal nodules (SENs) are hamartomas smaller than 1 cm situated between the striatum and ventricle near the V-SVZ.^45^ SENs occur in the vast majority of TSC patients.^46^ Histologically, they contain abundant DCX and Tuj1 positive immature neuroblasts dispersed within a glial fibrillary matrix along with sporadic NeuN, Iba1, Ki67, or Sox2/Pax6 positive cells.^47^ SENs can appear as early as ∼mid-gestation within the subependymal region and contain dysmorphic astrocyte-like cells that express vimentin, glutamine synthetase, GFAP, and filamin A.^48^ Embryonic SEN-like lesions and postnatal SENs also contain occasional giant cells, a pathognomonic feature of TSC.^47^^,^^48^ Given the early onset and anatomical location of SENs, the cellular composition is not surprising. In humans, V-SVZ NSCs are detectable throughout life although their ongoing division and generation of immature inhibitory neuroblasts declines to undetectable levels approximately 18 months after birth.^49^^,^^50^^,^^51^ In nearly 10–30% of TSC cases, patients have subependymal giant cell astrocytomas (SEGAs) which may originate from continued SEN growth.^46^ SEGAs are often located near the caudate nucleus of the striatum and often arise *in situ* near the parenchyma V-SVZ border, and without disruption of the ventricular, ependymal lining.^48^^,^^52^ SEGAs also contain giant cells that express markers found in dysmorphic astrocytes but have a conspicuous and eccentrically localized nuclei with prominent nucleoli, are eosinophilic and can be multi-nucleated.^53^ Giant balloon-like cells having glial-like features are also present in late fetal SEGA-like growths, followed by the appearance of small fusiform cells including migrating neuroblasts and inhibitory interneurons.^48^ Eventually, SEGAs will contain sporadic inhibitory interneurons and potentially BIII tubulin positive neuroblasts.^47^^,^^48^^,^^52^^,^^54^ Such neuroblasts and neurons could theoretically be generated by the cell or origin of SEGAs. Thus, a hypothesis is that mutations arising in V-SVZ NSCs might cause SEGAs. Nestin, GLAST, and SOX2 which NSCs express, also are found in SEGAs suggesting that they may contain and/or originate from NSCs.^47^^,^^55^ In agreement, RNA sequencing has revealed an increase in NSC specific transcripts in SEGAs.^56^^,^^57^^,^^58^^,^^59^ However, given the disease state, these transcripts could reflect aberrant transcription in non-NSC cells.

How loss of TSC genes causes brain hamartomas and effects NSC differentiation remains uncertain. In support of the hypothesis that loss of TSC genes in cells along the V-SVZ/striatal border causes subcortical hamartomas including SENs and SEGAs, nodules can be generated in mice by removing conditional *Tsc1* alleles from postnatal V-SVZ NSCs or progenitors.^60^^,^^61^ *Tsc1* knockout neuroprogenitors generate neuroblasts and neurons that are ectopically positioned.^60^^,^^61^ NSC proliferation and differentiation appear to be unaffected by loss of *Tsc1 in vitro.*^60^ In combination with loss of *Tsc1*, removal of *PTEN* from neonatal V-SVZ NSCs caused SEGA-like hamartomas which retain stem-like properties.^47^ Incidentally, loss of TSC genes in human organoid models appears to prevent neuronal differentiation.^62^ Yet inducible *Tsc1* knockout in human cortical organoids prevents neuronal but enhances glial production.^63^ Thus, some TSC anatomical alterations might reflect an important role for TSC genes in fate decisions including gliogenesis. In agreement, loss of TSC genes using a GFAP promoter driven CRE also increases the number of GFAP positive astrocytes.^35^^,^^64^^,^^65^^,^^66^ However, *TSC2* mutations are responsible for the majority of SEGAs and studies have only recently started to examine the role of *Tsc2* in V-SVZ NSCs and their capacity to generate different cell types.^67^^,^^68^

The following study utilizes a combination of *in vivo* genetic manipulation, whole transcriptome RNA-sequencing (RNA-seq), polysome profiling, and microscopy to determine what role, if any, *Tsc2* has in V-SVZ NSCs and differentiation. *Tsc2* mutant NSCs were confirmed to have increased mTOR pathway activity, altered translation factor mRNA, and stochastic mRNA translation. Changes in the amplitude and efficiency of translation were associated with the inability to down-regulate stem features in mutant *Tsc2* cells. We propose that TSC gene mutations prevent the loss of stemness which may have implications in developing novel therapeutic approaches to treat patients.

## Results

### Single cell V-SVZ NSC Tsc2 knockout generates hamartomas

**Figure 1:**
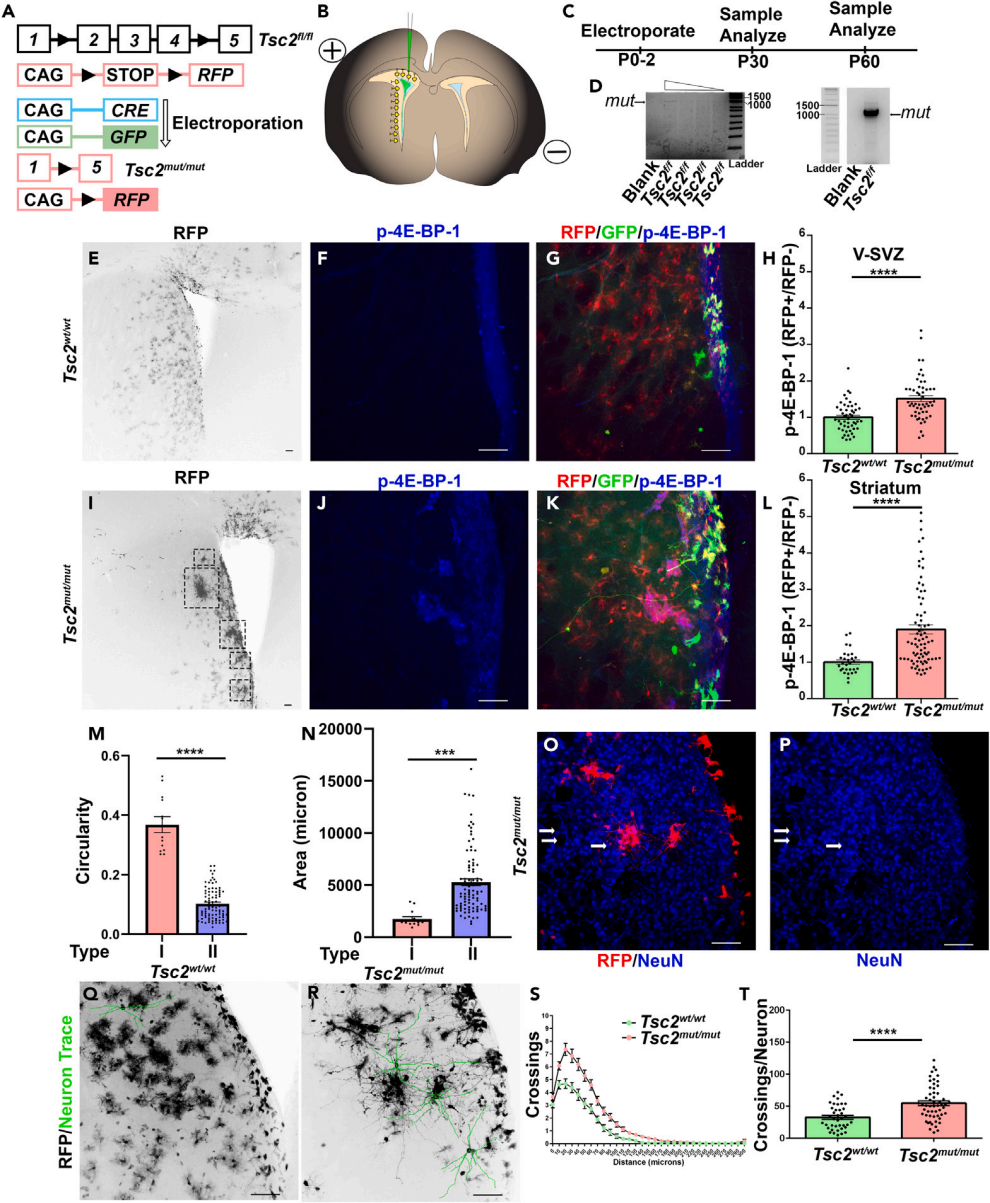
*NSC Tsc2 deletion generates striatal hamartomas* (A) Schematic diagram of conditional *Tsc2* and inducible *RFP* genes. *Tsc2* is mutated and RFP is expressed when CRE recombinase is present. (B) Schematic diagram of CAG-CRE and CAG-GFP (Green) plasmid electroporation which induces genomic CAG-RFP leading to co-expression (yellow). (C) Timeline of electroporation and analysis. (D) Long-range PCR of *Tsc2* wild-type and mutant alleles following electroporation of CRE-recombinase with varying starting amounts and low exposure to confirm precise size (left, approximately 1090 base pairs) and high exposure demonstrating efficient recombination (right). (E) 5x *Tsc2*^*w*^^*t*^^*/w*^^*t*^ coronal section demonstrating labeling along the V-SVZ, cortex, and striatum at P30. (F and G) 20x *Tsc2*^*w/w*^ coronal section stained for p4E-BP at P30. (H) Quantification of p4E-BP1 in the V-SVZ at P30. *Tsc2*^*w*^^*t*^^*/w*^^*t*^, N = 6, n = 49, mean = 1.00 ± 0.057 SEM vs. *Tsc2*^*mut/mut*^, N = 8, n = 53, mean = 1.51 ± 0.081 SEM. (I) *Tsc2*^*mut/mut*^ coronal sections demonstrating labeling along the V-SVZ, cortex, and striatum at P30. Dotted squares denote hamartomas. (J and K) 20x *Tsc2*^*mut/mut*^ coronal section stained for p4E-BP at P30. (L and M) Quantification of p4E-BP1 in the striatum at P30. *Tsc2*^*w*^^*t*^^*/w*^^*t*^, N = 6, n = 29, mean = 1.00 ± 0.06 SEM vs. *Tsc2*^*mut/mut*^, N = 8, n = 79, mean = 1.898 ± 0.013 SEM M) Quantification of hamartoma size *Type I*, N = 8, n = 13, mean 1,764 μm = 216.8 ± SEM vs. *Type II*, N = 16, n = 92, mean = 5,273 μm ± 333.7 SEM. (N) Quantification of hamartoma circularity. *Type I*, N = 8, n = 13, mean = 0.368 ± 0.027 SEM vs. *Type II*, N = 16, n = 92, mean 0.1022 = ± 0.005 SEM. (O and P) Example of Neu-N positive heterotopic neurons within striatal hamartomas at P30. (Q and R) Sample tracing showing cellular morphology. Note that traces in P are from the section shown in M-N. (S) Sholl analysis of *Tsc2*^*w*^^*t*^^*/w*^^*t*^ and *Tsc2*^*mut/mut*^ neurons at P30. (T) Total number of dendrite crossings at P30. *Tsc2*^*w*^^*t*^^*/w*^^*t*^, n = 39, mean = 32.64 ± 3.046 SEM vs. *Tsc2*^*mut/mut*^, n = 55, mean = 54.98 ± 3.682 SEM ∗∗∗ = p < 0.001, ∗∗∗∗ = p < 0.0001. Data are represented as mean ± SEM. Scale bar = 75 μm. See also Figures S1–S3.

Electroporation of Cre recombinase (CRE) and green fluorescent protein (GFP) plasmids into neonatal mice having wild-type (*wt*) or conditional (*f*) *Tsc2* alleles was performed to mimic somatic *Tsc2* biallelic inactivation (*mut*) and to generate a model of TSC hamartomas and brains analyzed at P30 and P60 (Figures 1A–1C). CRE induced RFP expression and *Tsc2* allele recombination (Figure 1D). *Tsc2*^*w*^^*t*^^*/w*^^*t*^ NSCs generated cells having a glial morphology that was confirmed by glutamine synthetase (GS) staining (Figure 1E). *Tsc2*^*w*^^*t*^^*/w*^^*t*^ glia had low mTOR pathway activity as indicated by eukaryotic initiation factor 4E binding protein (4E-BP) phosphorylation (Figures 1F and 1G). In contrast, *Tsc2*^*mut/mut*^ SVZ p4E-BP was elevated in comparison to controls (Figures 1H–1K). *Tsc2*^*mut/mut*^ NSCs also generated p4E-BP positive nodular hamartomas near the lateral V-SVZ and striatum (Figures 1I–1K; Figures S1A–S1S). Hamartomas had elevated p4E-BP in comparison to *Tsc2*^*w*^^*t*^^*/w*^^*t*^ striatal glia (Figures 1J–1L). Hamartomas could be categorized based on circularity which was measured by tracing the perimeter of lesions (Figure 1K). DNA counterstaining confirmed disorganized regions within the striatum (Figures S2A–S2R). 105 hamartomas were quantified having an average size of 4,834 μm^2^ (SEM = 314.508) and could be categorized as linear or round with round lesions being smaller (Figures 1M and 1N). Hamartomas frequently contained cytomegalic Neu-N positive neurons (Figures 1O and 1P). Neurons had hypertrophic dendrites with >7 primary dendrites originating from a cytomegalic soma with a round nucleus (Figures 1Q–1T). *Tsc2*^*w*^^*t*^^*/w*^^*t*^ striatum, in contrast, had fewer neurons (Figure S3A). *Tsc2*^*w*^^*t*^^*/w*^^*t*^ and *Tsc2*^*mut/mut*^ neurons were also found within the nucleus accumbens (Figures S3B and S3C) and olfactory bulb (OB) as expected.^69^^,^^70^ *Tsc2*^*mut/mut*^ striatal neurons were morphologically distinct from OB granule cells (GCs) and were larger than control striatal neurons (Figures S3D and S3E).^70^ Their morphology instead resembled neurons generated by reprogrammed striatal glia.^71^^,^^72^^,^^73^^,^^74^ *Tsc2*^*mut/mut*^ striatal neurons had more dendrites than *Tsc2*^*w*^^*t*^^*/w*^^*t*^ neurons (Figures 1S and 1T). The presence of more *Tsc2*^*mut/mut*^ neurons in the lateral striatum was unlikely caused by defective migration since the ratio of mutant neurons in each region did not change (Figures S3B and S3D). Taken together, NSC *Tsc2* deletion produced striatal hamartomas with heterotopic neurons.

### Loss of Tsc2 in V-SVZ NSC alters transcripts encoding translational regulatory factors

**Figure 2:**
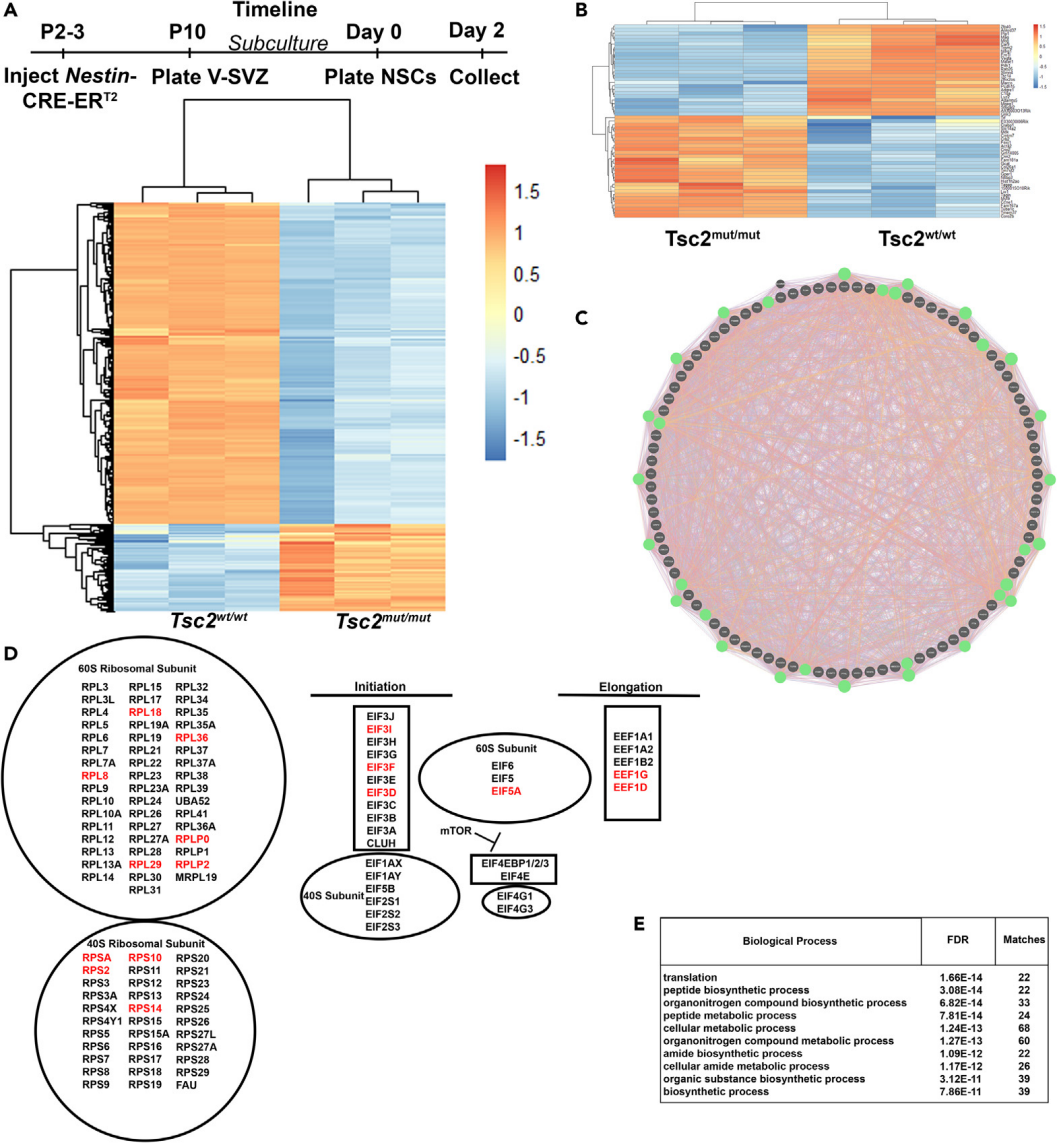
*NSC Tsc2 deletion alters ribosome biogenesis* (A) Upper-Timeline of NSC culturing and RNA sequencing. Lower-Heatmap and hierarchical clustering of mRNA transcripts from *Tsc2*^*w/w*^ and *Tsc2*^*mut/mut*^ NSCs. (B) Example of topmost increased and decreased mRNA transcripts from *Tsc2*^*w/w*^ and *Tsc2*^*mut/mut*^ NSCs. (C) Network analysis of differentially expressed transcripts from *Tsc2*^*w/w*^ and *Tsc2*^*mut/mut*^ NSCs. Green circles highlight proteins critical for translation. (D) Translation-related genes in top 100 most up-regulated genes by Tsc2 deficiency (red). (E) G.O. analysis indicating top biological processes represented in differentially expressed transcripts. See also Figures S4–S7.

V-SVZ *Tsc2*^*w*^^*t*^^*/w*^^*t*^ and *Tsc2*^*mut/mut*^ cells were subsequently cultured from *Tsc2*^*w*^^*t*^^*/w*^^*t*^ and *Tsc2*^*mut/mut*^ *x Nestin-*CRE-ER^T2^ neonatal mice and RNA sequencing performed to identify mechanisms that may be responsible for hamartoma and heterotopic neuron generation. *Tsc2*^*mut/mut*^ cultures contained cytomegalic cells with multinucleated giant cells (Figures S4A and S5). Multinucleated cells were also confirmed in the cerebral cortex and in dorsal astrocyte cultures (Figures S4B and S4C). Subculturing cells under NSC self-renewing conditions resulted in a homogeneous population of RFP positive NSCs (Figure S3A). *Tsc2*^*mut/mut*^ NSCs could self-renew slightly faster as monolayer cultures and could be grown as neurospheres in the absence of a substrate (Figures S6A–S6D). NSC subcultures were confirmed to have lost *Tsc2* (Figures S4D and S4E). Whole transcriptome bulk RNA sequencing of sub-cultured NSCs maintained under self-renewing conditions confirmed expression of canonical NSC mRNAs including *Vimentin*, *Olig1/2*, *TNC*, *Nestin*, and a battery of *Sox* genes. Several transcripts present in the proposed originating cells of TSC SEGAs including *Ttyh1*, *Ptgds*, *GFAP*, *GAD1*, *EGFR*, and *Ednrb* were also present.^52^ Hierarchical clustering categorized NSCs into two groups, *Tsc2*^*w*^^*t*^^*/w*^^*t*^ and *Tsc2*^*mut/mut*^ NSCs (Figure 2A). RNA-seq identified 2,268 differentially expressed mRNAs based upon a *P adjusted value* < 0.00001, and more than log (base 2) two-fold enrichment. Most transcripts were downregulated, and 571 transcripts were increased (Figure 2B). Bioinformatic analysis using GeneMANIA and the most abundant differentially expressed transcripts pointed to a network of altered translation factors (Figure 2C). Indeed, Cytoscape analysis indicated that protein translation pathways including initiation and elongation as well as ribosome encoding transcripts were altered (Figure 2D). Gene ontology (GO) analysis revealed enrichment of pathways related to ribosome biogenesis and metabolism (Figure 2E). However, ribosome proteins (rps3, rpl26, rpS6) were often altered independently of mRNA transcript levels as determined by western blot analysis of precipitated protein pellets from the same RNA isolations, indicating that post-transcriptional mechanisms may be altered (Figure S4D). Nevertheless, comparison of the differentially expressed transcripts revealed overlap with transcriptomic profiles from TSC SEGAs (Figures S7A–S7D).^57^^,^^75^^,^^76^ Taken together, *Tsc2* mutant NSCs had altered transcript levels of factors that regulate translation.

### Translational programs that encode for ribosome biogenesis, metabolism, and development are altered in Tsc2 knockout V-SVZ NSCs

**Figure 3:**
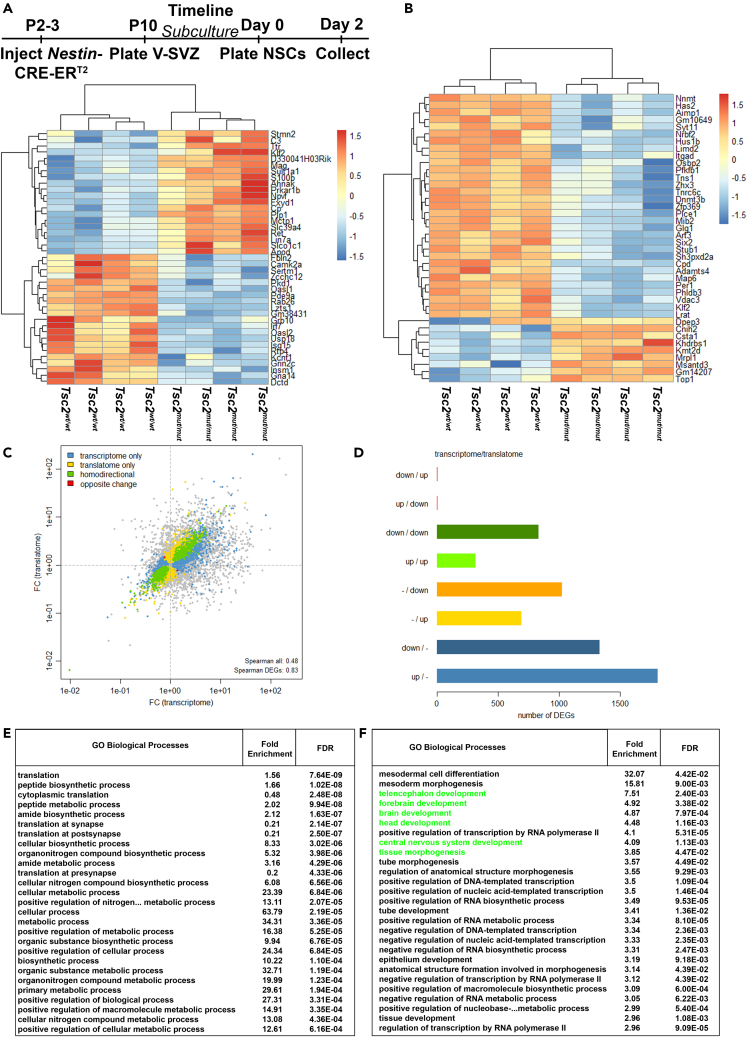
*NSC Tsc2 deletion alters mRNA translation dynamics* (A) Upper-Timeline of NSC culturing and RNA sequencing. Lower-Heatmap of topmost differentially translated polyribosomal mRNAs. (B) Heatmap of topmost differentially translated mRNAs. (C) Scatterplot of mRNA transcript availability compared to translatome abundance. Each diamond represents an mRNA transcript plotted on the x axis which represents the total mRNA abundance (transcriptome) and the y axis represents the polyribosome abundance (translatome). A gray diamond indicates there is no change between *Tsc2*^*w*^^*t*^^*/w*^^*t*^ and *Tsc2*^*mut/mut*^. A blue diamond indicates changes to the total mRNA abundance only without translational changes. A yellow diamond indicates changes only to the translatome. A green diamond denotes changes for both transcriptome and translatome that occur in the same direction (i.e., more total mRNA correlates with more polyribosome mRNA = homodirectional). A red diamond reflects changes in the opposite direction (more total abundance but less polyribosome abundance). (D) Histogram of transcript and translatome abundance and changes. (E) G.O. analysis of most highly expressed mRNAs with altered translation efficiency. (F) G.O. analysis of mRNAs having the most highly increased translational efficiency. Green highlights those terms mentioned in text.

To determine whether mRNA translation was altered, actively translating NSC mRNAs were subsequently identified by polyribosomal profiling. 628 transcripts were differentially recovered with polyribosomes, 316 of which were elevated following loss of *Tsc2* (Figure 3A, *p-adjusted value < 0.0001*). *CTGF*, which is increased in TSC patients and TSC mouse models, was among the top up-regulated genes.^77^ Inflammatory processes were also significantly differentially affected. This is consistent with the inflammatory and reactive states often detected within TSC malformations. The extent that mRNA within polyribosomes reflected total RNA abundance was unclear. Transcriptomes of translatome-paired samples were also quantified. The ratio of polyribosomal to total mRNA was then determined as a measure of translational efficiency (Figure 3B). Translatome (polyribosome) levels positively correlated with transcript availability (Figure 3C). Transcripts that are differentially translated in *Tsc2*^*mut/mut*^ NSCs were more tightly correlated with their transcript availability (Figure 3C). However, not all transcripts whose mRNA availability changed were differentially translated. Nevertheless, the translational efficiency of several transcripts was altered in *Tsc2*^*mut/mut*^ NSCs (Figure 3D). The most abundant transcripts with differential translational efficiency included those that regulate translation (Figure 3E). In addition, transcripts with the greatest increase in translational efficiency included those that regulate developmental processes including telencephalon, forebrain, brain, head, and central nervous system development as well as differentiation and morphogenesis (Figure 3F). Taken together, these results confirm aberrant translation regulation in TSC NSCs and indicate that NSC developmental events may also be impacted during hamartoma formation.

### Single cell Tsc2 knockout followed by single nuclei RNA sequencing reveals changes in translation regulatory and metabolic transcripts and NSC transition states

**Figure 4:**
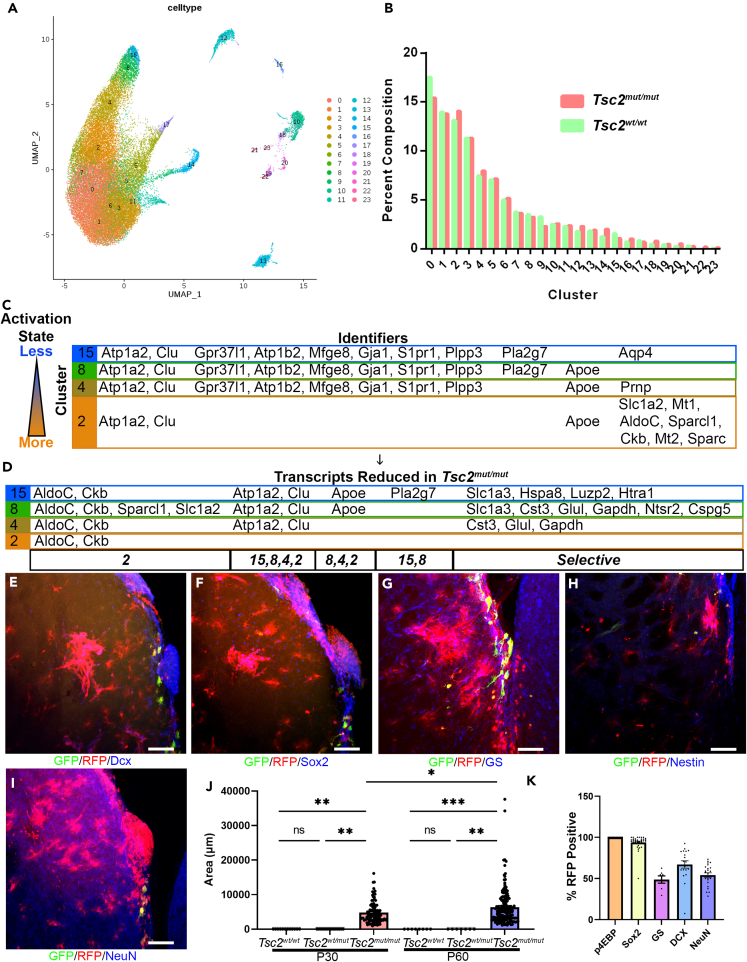
*Tsc2 mutant neuroprogenitors exhibit altered transitional states* (A) U-MAP clustering of V-SVZ microdissected cells from four mice for each genotype (*Tsc2*^*w*^^*t*^^*/w*^^*t*^ and *Tsc2*^*mut/mut*^) and represented as a composite. Data represent an N of 8 mice. (B) Percentage of cells represented in respective clusters. (C) Summary of top 10 transcripts (Identifiers) enriched in the indicated clusters 15, 8, 4, and 2. Each row indicates a cluster type and their representative activation states (left) with cluster 15 being the least activated and cluster 2 being the most activated. See Table S2 for additional clusters and respective differentially enriched transcripts. (D) Each row represents the indicated cluster (15, 8, 4, and 2). Each cluster (row) contains the differentially expressed transcripts in *Tsc2*^*mut/mut*^ mice compared to *Tsc2*^*w*^^*t*^^*/w*^^*t*^ mice. Each listed transcript is significantly decreased in the *Tsc2*^*mut/mut*^ condition. Note that the transcripts also happen to be important identifiers of clusters listed in C. For reference, cluster numbers were placed at the bottom of D to identify the clusters that the transcripts are identifiers of. The decrease in transcripts reflect changes in activation states, most notably, a reduction in transcripts found in cluster 2 indicating a change in activation dynamics. Columns represent the clusters that the transcripts are enriched in. (E–I) 20x images of CRE and GFP electroporated brains with *Tsc2*^*mut/mut*^ RFP (red) and DCX (E, blue), Sox2 (F, blue), Glutamine Synthetase (GS) (G, blue), Nestin (H, blue), or NeuN (I, blue) stained hamartomas and nodules. (J) Quantification of hamartoma size in *Tsc2*^*w*^^*t*^^*/w*^^*t*^*, Tsc2*^*wt/mut*^ and *Tsc2*^*mut/mut*^ mice at P30 and P60. (K) Quantification of cell markers in hamartomas. *p4E-BP*, N = 9, n = 24, mean = 100 ± 0 SEM; *DCX*, N = 7, n = 19, mean = 67.04 ± 4.496 SEM; *Sox2*, N = 11, n = 32, mean = 93.79 ± 1.618 SEM; *GS,* N = 4, n = 7, mean = 48.74 ± 4.429 SEM; *NeuN*, N = 9, n = 23, mean = 54.18 ± 2.722 SEM. N = mice, n = hamartoma. Data are represented as mean ± SEM. Scale bar = 75 μm for E-H and 7.5 μm for I-K. See also Figures S8 and S9.

Single nuclei RNA sequencing (snRNA-seq) of V-SVZs was performed to determine how altered *Tsc2*^*mut/mut*^ NSC transcriptomes and translatomes create hamartomas *in vivo*. Twenty-three cell clusters were identified (Figure 4A). Clusters 15, 8, 4, and 2 were mapped in graphical proximity based on cluster analysis thereby indicating similar identities (Figure 4A). Minor fluctuations in the percentage of clusters were noted, but *Tsc2*^*mut/mut*^ cells had similar cluster compositions to those of *Tsc2*^*w*^^*t*^^*/w*^^*t*^ conditions (Figure 4B). Cell clusters included NSCs denoted by shared markers *ATP1a2* and *Clu* as well as *Gpr37L1*, *Atp1b2*, *Mfge8*, *Gja1*, *S1pr1*, and *Plpp3* which were enriched in clusters 15, 8, and 4 (Figure 4C). Markers denoted a continuum of NSC activation states as previously reported with cluster 2 expressing *AldoC*, *Sparcl1*, *Sparc*, and *Ckb* which are characteristic of active NSCs (Figure 4C).^7^ Thus, as one might expect, some transcripts overlapped (Figure 4C). However, others were specific to activation state (Figure 4C). Additional clusters including astrocyte-like cells (Cluster 1–2) defined by higher *Slc1a2*, oligodendrocytes (Cluster 11 expressed *Plp1* and *Mbp*), and numerous neural lineages (7, 10, 13, 18–20) were identified (Table S1). Two lineages expressed *Ptgds*, a marker of recently described caudal late interneuron progenitor cells (Cluster 11, oligo and Cluster 5 unknown) (Table S1). Ependyma (Cluster 16, denoted by *Syne1*) were additionally identified (Table S1). *Tsc2*^*mut/mut*^ and *Tsc2*^*w*^^*t*^^*/w*^^*t*^ transcripts were subsequently compared between the same clusters, for example we compared cluster 2 between the two genotypes (Figure 4D). Transcripts that represented cluster 2 NSC activation states were reduced in *Tsc2*^*mut/mut*^ NSCs. For example, cluster 2 transcripts, Ckb and *AldoC*, were significantly downregulated in *Tsc2*^*mut/mut*^ NSCs compared to *Tsc2*^*w*^^*t*^^*/w*^^*t*^ NSCs (Figure 4D). *AldoC and Ckb* were in fact reduced in all *Tsc2*^*mut/mut*^ NSC states reflecting a change in dynamics of NSC transitional states. In agreement, *AldoC* was one of the first identified down-regulated transcripts in SEGAs.^56^ Moreover, *Sparcl1* down-regulation detected in snRNA-seq data were also confirmed in RNA-seq data as one of the most significantly down-regulated transcripts in cultured *Tsc2*^*mut/mut*^ NSCs. *Tsc2*^*mut/mut*^ NSCs had elevated *Rpl39*, *Rplp1*, and *Rps21* expression whereas primed/activated *Tsc2*^*mut/mut*^ NSC clusters had reduced ribosomal transcripts. Dysregulation of mRNA transcripts also became more evident as cells advanced toward a more differentiated trajectory (Table S2). Incidentally, approximately 1% of cells were identified as choroid plexus epithelial cells. However, a slightly larger percentage of cells in *Tsc2*^*mut/mut*^ conditions clustered in this group. *Tsc2*^*mut/mut*^ clusters up-regulated choroid plexus markers including *Ttr* which was also elevated in TSC transcriptomes (Figure 3A). Surprisingly, the *Tsc2*^*mut/mut*^ cluster 9 and choroid plexus cluster 14 had higher levels of *Ptgds*. *Ptgds* was previously identified as a marker of caudal late inhibitory progenitors implicated in the formation of TSC SEGAs. *Ptgds* is highly enriched in the choroid plexus as confirmed in the Allen Brain Atlas and MIT Single Cell Portal (data not shown). Nevertheless, snRNA-seq demonstrated that loss of *Tsc2* alters transcripts that identify unique NSC activation states.

The mTOR pathway and translation are tightly controlled during neurogenesis. Active NSCs have high mTOR activity and newly generated neuroblasts have lower mTOR activity.^78^ We also found that NSCs have higher mTOR pathway activity in comparison to striatal glia. In contrast, mTOR pathway activity was elevated in striatal hamartomas. Thus, controlling mTOR regulation of translation programs might be critical for the maintenance of stemness. One would therefore hypothesize that downregulation of mTOR and translation in glia might help uncouple stemness transcript availability from translation of stem factors. However, when *Tsc2* is mutated, stem factors may not be properly turned off. This could lead to ectopic generation of neurons as reported above. In support of the hypothesis that *Tsc2* is required for regulating neuroprogenitor-glia differentiation, hamartomas also contained newborn neuroblasts as indicated by the presence of Dcx (Figures 4E; Figures S8A and S8B). Moreover, hamartomas expressed and retained elevated Sox2, a marker of neuroprogenitors (Figures 4F; Figures S8C and S8D). Approximately half of all cells expressed glutamine synthetase confirming a glial component of hamartomas (Figures 4G; Figures S9A and S9B). Finally, hamartomas contained cells that expressed Nestin, which identifies NSCs (Figures 4H; Figures S9C and S9D). NeuN positive neurons were also enriched in striatal hamartomas (Figure 4I, Figures S9C, S9E, and S9F). *Tsc2*^*mut/mut*^ hamartomas were confirmed to slowly grow from P30 to P60 but were completely absent in *Tsc2*^*w/w*^ or *Tsc2*^*wt/mut*^ conditions (Figure 4J). In conclusion, hamartomas were characterized by elevated mTOR pathway activity, stem-like cells, and aberrant neurogenesis within the striatum (Figure 4K). Taken together, *Tsc2* loss of function alters translation through numerous mechanisms and alters cellular differentiation.

## Discussion

We have shown that *Tsc2* is required for neuroprogenitor differentiation. mTORC1 activity is meticulously regulated as V-SVZ neuroprogenitors transition from quiescent to active states and during neural differentiation.^78^^,^^79^^,^^80^^,^^81^ One potential reason for tightly controlling mTORC1 activity is the need to decouple mRNA availability and translation during the transition from stem states. Here it is demonstrated that lateral V-SVZ neuroprogenitors generate glia with repressed mTORC1 activity. The reduction of mTORC1 pathway activity in cortical astrocytes has been previously documented and the study here extends this finding to striatal astrocytes.^39^ We found that loss of *Tsc2* prevented the downregulation of mTORC1 activity in a subset of cells within the striatum. These cells were located within nodular growths. The heterogeneous cellular morphologies and hence composition led us to propose that these growths represent hamartomas that occur in the neurodevelopmental disorder TSC. TSC hamartomas include subependymal giant cell astrocytomas (SEGAs) that form along the striatum and V-SVZ. Occasional hamartomas appeared to protrude into or invade the ventricular wall. Hamartomas were characterized by abnormal heterotopic clusters of morphologically heterogeneous cells which included neurons. Hamartomas had characteristically elevated mTORC1 activity. Therefore, this model allowed us to examine the molecular and cellular underpinnings of brain hamartoma formation.

The presented study uncovered a transcriptional signature of dysregulated translational components in *Tsc2* null NSCs. Transcriptional changes and the cellular morphology of NSCs was similar to a seminal finding that proposed mice could be used to model human TSC giant cells.^82^ That model demonstrated that homozygous but not heterozygous *Tsc2* mutant NSCs from mice have robust transcriptional changes and defects in neural differentiation.^82^ Subsequent studies used conditional *Tsc1* deletion in embryonic NSCs (*GFAP, Emx1*, and *Nestin* promoter driven CRE) which caused cytomegaly, macrocephaly, hypomyelination, and gliosis with seizures.^35^^,^^37^^,^^38^^,^^83^ *hGFAP*-CRE *Tsc2* deletion produced subtle effects on dorsal NSCs but was reported to increase GFAP in the V-SVZ which may reflect the presence of striatal hamartomas as reported here.^35^ *Tsc1* deletion by *Emx1*-Cre also increases *Pax6* NSCs and BrdU labeling, but leads to NSC exhaustion, and increased GFAP positive cells.^38^ BrdU labeling at E15.5 resulted in fewer BrdU positive Ki67 negative cells in the *Emx1-Cre* x *Tsc1* model and the authors hypothesized this may indicate abnormal differentiation.^38^ In a congruent line of thought, several groups have proposed that mTORC1 regulation of translation may be important for V-SVZ NSCs. For example, mTORC1 was shown to be active in transit amplifying cells (TACs) but not in NSCs and that inhibition of mTORC1 causes a reversible quiescent phenotype that depletes TACs.^79^ However, a conflicting report indicated that dividing NSCs also have active mTORC1 which is required for TAC generation and that up-regulation of mTORC1 activity through 4E-BP1 phosphorylation induces differentiation.^80^ On the other hand, expression of constitutively active mTOR in NSCs induces massive Hif1a dependent apoptosis.^84^ Nevertheless, these have focused on dorsal NSCs because of their relationship to cortical development and cortical tubers which may not be applicable to postnatal lateral and ventral V-SVZ NSCs.

The role of V-SVZ NSCs in TSC has been previously examined in rodents as they may be a cell of origin for neurodevelopmental diseases and brain tumors which stems from the fact that brain growths are often highly heterogeneous.^85^ Single-cell *Tsc1* deletion from NSCs was used to test the hypothesis that V-SVZ inhibitory NSCs generate SENs and SEGAs.^61^ Similar experiments used the same approach of conditional *Tsc1* mice crossed to tamoxifen-inducible *Nestin*-CRE or *Ascl1*-CRE mice.^60^*Tsc1* deletion caused nodules to form. Nodules had minor increases in pS6 and neuronal heterotopias. However, SEGAs were not reported. Homozygous deletion of a second gene, *Pten*, along with *Tsc1*, in neonatal NSCs, generated SEGA-like lesions.^47^ The *Pten/Tsc1* deletion model faithfully recapitulates most aspects of SEGAs and provides one of the most compelling examples that rodents can be used to model human brain disorders. However, whether the requirement for *Pten* deletion represents a technical hurdle to using mice or reflects a yet to be discovered genetic link to SEGAs is unclear. The model presented here differs in that only *Tsc2* was removed and has the additional advantages of low mortality, no seizure activity allowing for distinguishing seizure-dependent and seizure-independent changes, and focal hamartomas which mimics that seen in patients and therefore affords the tracking of cells and delineation of cell autonomous and intercellular effects of loss of *Tsc2*. Nevertheless, these results argue that rodents can generate hamartoma-like lesions and that deletion of TSC genes in neonatal V-SVZ NSCs can sufficiently model TSC.

Hamartomas in the model described here contained neurons having a striatal-like morphology which vastly differed from GC-like morphologies of the OB. Occasional granule cell-like neurons were present in the rostral migratory stream (RMS) and V-SVZ and could represent those destined for the OB. In agreement, neurons are scattered along the RMS, mirroring results using *Tsc1* or *Pten* V-SVZ deletion.^61^^,^^86^ However, neurons in the striatum in the current report had large dendrite arbors that differ greatly from *Tsc2*^*mut/mut*^ OB GCs.^70^ It is a plausible hypothesis that micro-hamartomas not detected by conventional clinical imaging techniques could contribute to TSC-associated neurological disorders.^44^ Given the location within the striatum, it could be that TSC neurons could modulate motor activity or reward pathways.^87^ Notably, balloon-like giant cells that were multinucleated and cytomegalic were also identified within the striatum. These cells were slightly Neu-N positive with moderate pS6 staining. Similar cells were occasionally detected within the cerebral cortex. These cells represent, in our opinion, the most compelling case that loss of *Tsc2* in murine models recapitulates TSC-specific patient cellular phenotypes. It is noteworthy that giant cells were often found among clusters of cells with astrocyte morphology. Postnatal striatal neurogenesis is reported by several groups and appears to be induced by a wide range of pathophysiological conditions as occurs in stroke or supraphysiological conditions as demonstrated by reprogramming of striatal astrocytes.^71^^,^^73^^,^^74^^,^^88^^,^^89^^,^^90^^,^^91^ Notch signaling appears to be critical in reprogramming striatal astrocytes.^90^ Notch signaling is altered in TSC.^92^^,^^93^ Indeed, loss of *Tsc1* has been shown to alter Notch and alter the progression of NSC-like cells in lymphangioleiomyomatosis and along the neural tube.^93^ Thus, loss of *TSC* genes leading to hyperactive mTORC1 might be hypothesized to prevent NSC-astrocyte programming. Most patient therapies are directed toward inhibiting mTORC1 and alleviating neurological symptoms. However, promoting terminal differentiation could in theory reduce the growth and development of TSC hamartomas.

V-SVZs were cultured, and cytomegalic multinucleated giant cells were found adhering to culture plates thereby supporting *in vivo* observations of giant cells. *Tsc2*^*w*^^*t*^^*/w*^^*t*^ vs. *Tsc2*^*mut/mut*^ NSCs had consistent transcriptomic differences. Notable differences included changes in translational regulatory and ribosome encoding transcripts. However, attempts to confirm changes at the protein level were often fruitless with unpredictable changes. We hypothesized that stochastic changes in mRNA translation caused by mTORC1 phosphorylation of translational machinery might be involved. Thus, delineating whether transcriptomic changes are a direct effect of loss of *Tsc2* or secondary to changes in cellular homeostasis was not feasible in this study, although future studies could answer such a question by treating cells with mTORC1 inhibitors and examining consequences.

Changes to p4E-BP, pS6, and ribosome transcripts and ribosome proteins supported that translation is altered in *Tsc2*^*mut/mut*^ NSCs. Indeed, a unique translatome was confirmed by polyribosomal profiling. However, this in theory could reflect changes in the abundance of the total mRNA pool. Yet, when the total mRNA availability was corrected for, notable differences in translational efficiency were found. These results support the notion that the translational efficiency of specific transcripts is altered. Interestingly, translational efficiency of mitochondrial respiration and of translational regulators themselves were noted. This is consistent with the well-established role of TSC-mTOR pathway in controlling translation and specifically in regulating mitochondrial transcript translation. Thus, translation is subject to multi-tier regulation by *Tsc2*.

The capacity for TSC1/2 regulation of NSC differentiation appears conserved in human NSCs. Organoids that were genetically engineered to lose one or both alleles of *TSC1* or *TSC2* produced more astrocytes at the expense of neurons.^63^ However, loss of both alleles was required in this study. A recent manuscript described *Tsc2* heterozygous organoids generated from passage 40–50 induced pluripotent stem cells.^52^ Organoids developed small protrusions after 220 days in culture. Protrusions had cells with mTOR pathway activity, EGF receptor expression, and a high proliferative index. ScRNA-seq of organoids provided evidence that protrusions shared markers of caudal late gestation inhibitory interneuron progenitors (CLIPs). The CLIP cell origin differs from bulk sequencing of RNA from human SEGAs. Transcriptomic analysis of bulk RNA preparations support that SEGAs originate from and comprise mutant NSCs.^56^^,^^57^^,^^58^^,^^59^ Microarrays identified ∼50 differentially expressed transcripts in SEGAs including a reduction in *AldoC* which were reduced in our model.^56^ Another study used bulk RNA-seq of SEGAs and found 3,693 differentially expressed transcripts which included those found by microarray.^57^ Cellular deconvolution software identified immune cell transcripts as highly differentially expressed supporting the need to perform single cell resolution sequencing. SENs and SEGAs were enriched in immature neuron, NSC, and astrocyte markers. Analysis of an additional 19 SEGAs revealed that ∼10,000 transcripts were differentially expressed (4,621 increased and 4,779 decreased).^58^ ∼1,000 of these transcripts were shared between these RNA-seq studies. Differentially expressed transcripts include *Vimentin*, *Olig1*, *Olig2*, *Nestin*, *Sox* genes, *Ednrb*, *Egfr*, *Ttyh1*, *Ptgds*, *Nkx6-*, and *Nkx2*, *Hopx*, *Emx2*, *Prox1*, *Pdgfra*, and *Nr2f1* (*Coup-TFI*) indicating the presence of NSCs. However, *GFAP*, *SCGN,* or *Coup-TFII* were not. These latter transcripts are critical because their absence suggests a lateral or medial ganglionic eminence origin. A lateral or medial ganglionic eminence origin differs from the report that cultured TSC organoids and concluded a human specific caudal ganglionic eminence origin.^52^ Moreover, the *Pten-Tsc1* deletion mouse model provides strong support for the origin of SEGA-like lesions from V-SVZ NSCs.^47^ In addition, a more recent manuscript indicated that *Tsc2* knockout using *NKX2.1* CRE resulted in SVZ abnormalities consistently in the ventral SVZ and that this is because the ventral SVZ NSCs have more mTOR activity.^68^ The *NKX2.1* CRE is expressed in a wide range of progenitors beginning at embryonic day 10.5 and effects cells that produce inhibitory neurons within the pallidal telencephalon including the medial ganglionic eminence and OB. Taken together, these results support that an NSC-like cell originating from the lateral or medial ganglionic eminences is enriched in SEGAs. Nevertheless, given the contradictory reports in clinical literature of markers and cell types, it could be that there are multiple paths to forming a SEGA depending upon the originating cell type mutation.

SnRNA-sequencing of our mouse model confirmed NSCs as a cohort of cells that could be parcellated by activation state. Our findings highly overlapped with and hence were inspired by analysis of Troy positive V-SVZ NSCs.^7^ The preparations of our samples, although enriched in NSCs, also included a wide range of additional cell types which were also altered. Whether the changes reflect cell-autonomous or non-cell autonomous effects will require further experimentation. Nevertheless, loss of *Tsc2* decreased many NSC activation markers. *Tsc2* loss could cause precocious differentiation into other cell types. Alternatively, *Tsc2* loss could delay differentiation through the retention of stem cell transcripts. Hamartomas retained stem proteins in support of the latter hypothesis. In conclusion, these results prove that stemness is not lost and therefore differentiation is incomplete following V-SVZ NSC *Tsc2* deletion. These results demonstrate the evolutionarily conserved importance of *Tsc2* in regulating neuroprogenitor differentiation.

### Limitations of the study

The study reported here demonstrates the importance of *Tsc2* in differentiation. While it is tempting to generalize these findings to TSC, the applicability is less certain. Mouse models generated by conditional deletion of *Tsc1* or *Tsc2* have contributed greatly to our understanding of TSC pathogenesis including with regards to brain, heart, and kidney manifestations. However, despite the evolutionary conservation of the biochemical pathways, there remain considerable gaps in our knowledge in how TSC pathogenesis occurs *in vivo* in patients and the applicability of these rodent models. Models that use homozygous loss of function mutations appear appropriate since over 70% of mutations reported lead to loss of TSC protein expression and loss of heterozygosity occurs in nearly all growths. Despite detailed histopathological characterization and use of bulk techniques such as RNA-seq to determine the composition of human TSC SEGAs, the cellular composition remains unclear. While expansion of the extracellular matrix, immune infiltration, and calcification are well documented which could be sufficient to cause growths, whether additional changes in the cellular composition also occurs is unknown. In the absence of consensus for the composition and hence cellular origins, what the appropriate control is for TSC SEGAs is unclear. This has caused extraordinary challenges in comparing SEGA characteristics. For example, SEGAs often are found within lateral ventricles indicating they could arise from the choroid plexus. Should one therefore compare SEGA data to control choroid plexus is unclear. Whether loss of TSC genes in NSCs, as occurs in our model, reflects the *bona fide* cell of origin in patients is currently unanswerable. This is particularly relevant because of the distinct origins of another lesion in TSC patients called lymphangioleiomyomatosis. Moreover, the literature has conflicting histopathological characterization of the cell types in SEGAs.^19^ One potential reason for these differences is that many cell types may be able to generate SEGAs. SEGA cellular heterogeneity therefore might reflect ontogenetic heterogeneity. Indeed, while SEGAs are most frequently surgically removed from the foramen of Monro near the subfornical organ, SEGAs and SENs frequently initiate along other parts of the walls of the lateral ventricles including striatum.

Another limitation to the study presented here is that cells were cultured in defined media components. These conditions include exposure to exogenous ligands such as EGF. Cell culture conditions are highly unlikely to reflect *in vivo* conditions where factors such as EGF are released in gradients during specific time periods. Moreover, SEGAs are not aggressive malignancies and do not grow at excessively fast rates. Thus, pushing NSCs into a state of cell division would appear to have limited applicability. Nevertheless, given the protracted rate of human brain development, it could be argued that the events identified in the current study mimic events of early TSC hamartoma formation when NSCs are known to divide. Finally, technical aspects of the genetic manipulations performed remain a limitation to our study. The current study removed both TSC genes at the same time. This contrasts to the many patients born with one functional and one mutant allele. Thus, the complex interplay between heterozygous and homozygous mutant cells is not modeled. Nevertheless, the genetic manipulation does mimic what can occur in mosaic TSC patients and non-TSC patients that have brain lesions caused by TSC mutations which include SEGAs as well as focal cortical dysplasia.

## STAR★Methods

### Key resources table

**Table:** 

REAGENT or RESOURCE	SOURCE	IDENTIFIER
**antibodies**		

Goat Polyclonal Doublecortin	Santa Cruz Biotechnology	Cat#sc-8066; RRID: AB_2088494; Clone: C-18
Mouse Monoclonal Doublecortin	Santa Cruz Biotechnology	Cat#sc-271390; Clone: E−6
Rat Monoclonal Sox2	Invitrogen	Cat#14-9811-82; RRID: AB_11219471; Clone: Btjce
Rabbit Monoclonal pS6 Ser 240/244	Cell Signaling Technology	Cat#4838; RRID: AB_659977; Clone: 61H9
Rabbit Monoclonal p4EBP Thr 37/46	Cell Signaling Technology	Cat#2855; RRID: AB_560835; Clone: 236B4
Chicken Polyclonal Nestin	Novus Biologicals	Cat#NB100-1604; RRID: AB_2282642
Rabbit Polyclonal Glutamine Synthetase	Sigma Aldrich	Cat#G2781; RRID: AB_259853
Mouse Monoclonal NeuN	Sigma Aldrich	Cat#MAB377; RRID: AB_2298772; Clone: A60
Rabbit Monoclonal Tuberin	Cell Signaling Technology	Cat#4308; RRID: AB_10547134; Clone: D93F12
Rabbit Monoclonal ATF4	Cell Signaling Technology	Cat#11815; RRID: AB_2616025; Clone: D4B8
Rabbit Monoclonal Actin	Cell Signaling Technology	Cat#4970; RRID: AB_2223172; Clone: 13E5
Rabbit Monoclonal *p*-RPS6	Cell Signaling Technology	Cat#5364; RRID: AB_10694233; Clone: D68F8
Rabbit Monoclonal RPL26	Cell Signaling Technology	Cat#5400; RRID: AB_10698750; Clone: D8F6
Rabbit Monoclonal RPS3	Cell Signaling Technology	Cat#9538; RRID: AB_10622028; Clone: D50G7
Rabbit Monoclonal RPS6	Cell Signaling Technology	Cat#2217; RRID: AB_331355; Clone: 5G10

**Chemicals, peptides, and recombinant proteins**		

Euthasol	Virbac	Cat#78059296
(Z)-4-Hydroxytamoxifen	Sigma Aldrich	Cat#H7904
EdU	Invitrogen	Cat#C10337
TRIzol Reagent	Invitrogen	Cat#15596026
TRIzol LS Reagent	Ambion	Cat#10296010

**Critical commercial assays**		

NEBNext Ultra II RNA Library Prep Kit for Illumina	New England Biolabs Inc	Cat#E7770S
NEBNext Poly(A) mRNA Magnetic Isolation Module	New England Biolabs Inc	Cat#E7490S
NEBNext Multiplex Oligos for Illumina (Dual Index Primers Set 1)	New England Biolabs Inc	Cat#E7600S
10X Genomics Single Cell 3′ kit v3.1	10X Genomics	Cat#1000269

**Deposited data**		

Polysomal Profiling Sequencing Data	This paper	GSE229276, https://www.ncbi.nlm.nih.gov/geo/query/acc.cgi?acc=GSE229552
Total RNA Sequencing Data	This paper	GSE229552, https://www.ncbi.nlm.nih.gov/geo/query/acc.cgi?acc=GSE229276
snRNA Sequencing Data	This paper	GSE229549, https://www.ncbi.nlm.nih.gov/geo/query/acc.cgi?acc=GSE229549
Reference Mouse Genome GRCm38 (mm10)	Genome Reference Consortium	https://www.ncbi.nlm.nih.gov/datasets/genome/GCF_000001635.20/
Western Blots	This paper	https://doi.org/10.17632/sfspkt88tk.1, https://data.mendeley.com/datasets/sfspkt88tk/1

**Experimental models: Organisms/strains**		

Mouse: RFP^+/−^, RFP^+/+^: B6.Cg-Gt(ROSA)26Sortm9(CAG-tdTomato)^Hze/J^	Jackson Laboratories	Cat#007909; RRID: IMSR_JAX:007909
Mouse: Nestin-CRE-ER^T2^: C57BL/6-Tg(Nes-cre/ERT2)^KEisc/J^	Jackson Laboratories	Cat#016261; RRID: IMSR_JAX:016261
Mouse: Tsc2^wt/wt^, Tsc2^mut/mut^: Tsc2^tm1.1Mjg/J^	Jackson Laboratories	Cat#027458; RRID: IMSR_JAX:027458

**Oligonucleotides**		

Primers: *Tsc2:* 5′-ACAATGGGAGGCACATTACC-3′, 5- AAG CAGCAGGTCTGCAGTG-3′	Integrated DNA Technologies	N/A
Primers: RFP: 5′-AAGGGAGCTGCAGTGGAG TA-3′, 5′-CCG AAAATCTGTGGGAAG TC-3′, 5′- GGCATTAAAGCAGCGTA TCC-3’, 5′-CTGTTCCTGTACGGCATGG-3′	Integrated DNA Technologies	N/A
Primers: *Nestin-CRE-ER*^*T2*^: 5′-ATGCAGGCAAATTTTGG TGT-3′, 5′-CGCCGCTACTTCTTTTCAAC-3′, 5′-CTAGGCCA CAGAATTGAAAGATCT-3’ (Internal Positive Control), 5′- GTAGGTGGAAATTCTAGCATCATCC-3’ (Internal Positive Control)	Integrated DNA Technologies	N/A
Primers: *Nestin-CRE-ER*^*T2*^: 5′-ATACCGGAGATCATGCA AGC-3′, 5′-GGCCAGGCTGTTCTTCTTAG-3′, 5′-CTAGGCC ACAGAATTGAAAGATCT-3’ (Internal Positive Control), 5′- GTAGGTGGAAATTCTAGCATCATCC-3’ (Internal Positive Control)	Integrated DNA Technologies	N/A

**Recombinant DNA**		

Plasmid: CAG-CRE	Matsuda, T., and Cepko, C.L 2007.^94^	Cat#13775; RRID: Addgene_13775
Plasmid: CAG-GFP	Matsuda, T., and Cepko, C.L 2004.^95^	Cat#11150; RRID: Addgene_11150

**Software and algorithms**		

FIJI (ImageJ 1.5g)	Schindelin, J.et al.^96^	https://ImageJ.net/software/fiji/downloads
GraphPad Prism (v. 8.2.0)	GraphPad Software Inc	https://www.graphpad.com/
Sequencing Bioinformatic Analysis	DOI; https://doi.org/10.5281/zenodo.10066647	https://github.com/vshanka23/tsc_scrna_feliciano
FastQ Toolkit (v. 2.2.5)	BaseSpace (Illumina)	https://www.illumina.com/products/by-type/informatics-products/basespace-sequence-hub/apps/fastq-toolkit.html
RNA-Seq Alignment (STAR, v.2.6.1A)	BaseSpace (Illumina)	https://www.illumina.com/products/by-type/informatics-products/basespace-sequence-hub/apps/rna-seq-alignment.html
RNA-Seq Differential Expression (DESeq2, v.1.0.1)	BaseSpace (Illumina)	https://www.illumina.com/products/by-type/informatics-products/basespace-sequence-hub/apps/rna-seq-differential-expression.html
GeneSCF	Ashburner, M.et al.^97^^,^Carbon, S.et al.^98^	N/A
GeneMania in Cytoscape 3.60	Warde-Farley, D.et al.^99^	https://apps.cytoscape.org/apps/genemania
R-package: Pheatmap	Kolde, R^100^	https://cran.r-project.org/web/packages/pheatmap/index.html
R-package: tRanslatome	Tebaldi, T.^101^	https://bioconductor.org/packages/tRanslatome/
R-package: Seurat	Stuart, T.^102^	https://cran.r-project.org/web/packages/Seurat/index.html

### Resource availability

#### Lead contact

Further information and requests for resources and reagents should be directed to and will be fulfilled by the lead contact, David M. Feliciano (dfelici@clemson.edu).

#### Materials availability statement

This study did not generate new unique reagents.

#### Data and code availability

• Data. Single nuclei RNA, total transcriptome, and polyribosomal sequencing data have been deposited at the National Center for Biotechnology Information Gene Expression Omnibus (GEO) and are publicly available as of the date of publication. GEO identifiers are GSE229276, GSE229549, and GSE229552 as listed under the key resources table. URLs for downloading are provided in the key resources table. Data are provided as Fastq (bulk and polysomal sequencing) or.tsv and.mtx files. All metadata are provided as.csv files with the respective submission.
  Original Western blot images have been deposited at Mendeley Data as indicated in the key resources table and are publicly available as of the date of publication. The DOI is listed in the key resources table.
  Microscopy data reported in this paper will be shared by the lead contact upon request.
• All code is from published resources listed in the key resources table and additional details are provided at Github and are publicly available as of the date of publication. DOIs are listed in the key resources table.
• Any additional information required to reanalyze the data reported in this work paper is available from the lead contact upon request.

### Experimental model and subject details

#### Animals

All experiments conform to the relevant regulatory standards and were approved by the Clemson University Institutional Animal Care and Use Committee and the Animal Care and Use Review Office (ACURO), a component of the USAMRDC Office of Research Protections (ORP) within the Department of Defense (DoD). Red fluorescent protein (RFP^+/−^,^+/+^) (B6.Cg-Gt(ROSA)26Sortm9(CAG-tdTomato)^Hze/J^) (Strain #007909, RRID:IMSR_JAX:007909), C57BL/6-Tg(Nes-cre/ERT2)^KEisc/J^ (Strain #016261, RRID:IMSR_JAX:016261),^103^ Tsc2^tm1.1Mjg/J^ (Strain #027458, RRID:IMSR_JAX:027458)^104^ were acquired from Jackson Laboratories and were pathogen free. Sentinel mice were free of pathogens throughout the study. Sample size estimation was calculated using power analysis. Samples/subjects were allocated randomly to experimental group based on genotypes which the investigator was blinded to. Experimental manipulations were performed on mouse pups that were not involved in previous procedures and sacrificed accordingly. Ages and both sexes were used as indicated in figures. Mice were housed under standard pathogen-free conditions in cages on racks within isolated cubicles with a 12-h light/dark cycle and fed *ad libitum*. Experimental pups were housed with littermates and respective dams in microisolator cages following manipulation and were weaned at appropriate ages. Neonatal pups were then electroporated or injected with tamoxifen and sacrificed as described under method details. Weaned mice were housed with same sex littermates and sacrificed at the age indicated.

#### Primary NSC culture

B6.Cg-Gt(ROSA)26Sortm9(CAG-tdTomato)^Hze/J^) x C57BL/6-Tg(Nes-cre/ERT2)^KEisc/J^ and control or Tsc2^tm1.1Mjg/J^ mouse pups described above were injected with tamoxifen. Seven days after injections, mice were sacrificed and brains were bisected into two sagittal hemispheres. The V-SVZ was micro-dissected in ice-cold Neurobasal A (Gibco, #10888022) medium. 100 μL 0.25% Trypsin (Gibco, # 25200072) was added and incubated for 3 min at 37°C before the tissue was dissociated using three Pasteur pipettes having progressively decreasing bore sizes. 100 μL defined trypsin inhibitor (Gibco, #R007100) containing 0.01 mg/mL DNase1 was added, and the solution was centrifuged at 300 × g in an Eppendorf 5425 centrifuge. The pellet was resuspended in complete growth media (Neurobasal A (Gibco, #10888022), 0.02% Mouse FGF-basic Recombinant protein (Invitrogen, #RP-8626), 0.02% Recombinant Mouse EGF (Gibco, #PMG8044), 0.5% Pen Strep (Gibco, #15140-122), 0.008% 25 mg/mL Heparin sodium salt (Sigma Aldrich, #H3393), 1% Glutamax (Gibco, #35050-061), 2% B-27 Supplement without vitamin A (Gibco, #12587) before plating in a 100 mm dish coated with natural mouse Laminin (Gibco, 23017) and poly-D-lysine (Gibco, #A38904-01). Cells were cultured at 37 °C at 5% CO_2_ in a Isotemp water jacketed CO_2_ incubator (Fisher Scientific). Upon reaching confluency, cells were passaged by incubating in 5 mL Accutase (Invitrogen, #00-4555-56) for 5 min at 37°C. The cells were then spun down for 5 min at 300 × g in an Eppendorf 5425 centrifuge and replated at an appropriate density.

### Method details

#### Polymerase chain reaction (PCR)

Tissue was incubated in 50 mM NaOH and 0.2 mM EDTA at 50°C overnight. An equal volume of 100 mM Tris-HCl was added to samples. Samples were subject to routine genotyping using Taq DNA Polymerase with the following conditions: Initial denaturation step at 98°C for 2 min, followed by 35 cycles of denaturation at 95°C for 30 s, an annealing step at 60°C for 30 s, and an extension at 72°C for 30 s followed by a final extension at 72°C for 3 min. Samples were loaded onto a 1.7% agarose gel with 1X Blue Juice and run at 100 V for 20–30 mins. Mice having conditional *Tsc2* alleles are distinguished by endpoint genotyping PCR using the following primer sequences, 5′-ACAATGGGAGGCACATTACC-3′ and 5-AAGCAGCAGGTCTGCAGTG-3’. Tomato (RFP) genes were identified for Stock #7914 using the following primer sequences, 5′-AAGGGAGCTGCAGTGGAG TA-3′ and 5′-CCGAAAATCTGTGGGAAG TC-3′ and 5′-GGCATTAAAGCAGCGTATCC-3′ and 5′-CTGTTCCTGTACGGCATGG-3’. *Nestin-CRE-ER*^*T2*^ mice were genotyped with 5′-ATGCAGGCAAATTTTGGTGT-3′ and 5′-CGCCGCTACTTCTTTTCAAC-3′ or 5′-ATACCGGAGATCATGCAAGC-3′ and 5′-GGCCAGGCTGTTCTTCTTAG-3′ and 5′-CTAGGCCACAGAATTGAAAGATCT-3’ (Internal Positive Control) and 5′-GTAGGTGGAAATTCTAGCATCATCC-3’ (Internal Positive Control). Long-range PCR was performed as we previously described.^70^

#### Electroporation and tamoxifen injections

Neonatal mice were electroporated as previously described.^105^ Mouse pups were injected with equal concentrations and volumes of DNA plasmids diluted in phosphate buffered saline (PBS) with 0.1% fast green. Electroporated plasmids are as follows; CAG-CRE (Plasmid #13775, Addgene) and CAG-GFP (Plasmid #11150, Addgene).^94^^,^^95^ DNA was injected into the lateral ventricles and delivered using a borosilicate glass micropipette generated from pulled capillary tubes. Borosilicate capillary tubes were pulled with a P97 Sutter micropipette puller. Tweezer electrodes (model 520; BTX) were rinsed in 0.9% saline solution and swept over the head of neonatal pups using five, 100-volt square pulses of 50 ms duration with 950-ms intervals that were applied using a pulse generator (ECM830; BTX). *Nestin*-*CRE-ER*^*T2*^ mouse pups were weighed and injected with approximately 20 μg/g (Z)-4-Hydroxytamoxifen (Sigma Aldrich, #H7904) and 2.2 μg/g EdU (Invitrogen, #C10337).

#### Immunohistochemistry

Euthasol was administered by intraperitoneal injection to sedate mice followed by swift decapitation. Brains were dissected in room temperature PBS, transferred to 4% paraformaldehyde (in PBS), and incubated overnight at 4°C. Brains were rinsed in PBS and mounted in 3% agarose. A Leica VTS 1000 vibratome was used to slice brains coronally in 150–300 μm sections. Sections were blocked in PBS containing 0.1% Triton X-100, 0.1% Tween 20 and 2% BSA for 1 h at room temperature. Sections were washed in PBS containing 0.1% Tween 20 three times. Sections were incubated in primary antibody; anti-Dcx (1:500; Santa Cruz Biotechnology sc-8066 and sc-271390), anti-Sox2 (1:500; Invitrogen 14-9811-82) anti-pS6 (1:500; Cell Signaling Technology; Ser 240/244, 61H9, #4838), anti-Nestin (1:500; Novus Biologicals; #NB100-1604), anti-p4EBP (1:500; Cell Signaling Technology; Thr37/46, 236B4; #2855), and anti-glutamine synthetase (1:500; Sigma Aldrich; #G2781), anti-NeuN (1:500; Sigma Aldrich; #MAB377) overnight at 4°C. Sections were subjected to three additional washes in PBS containing 0.1% Tween 20. Sections were incubated with the appropriate secondary antibody (Alexa Fluor series; 1:500; Invitrogen) overnight at 4°C. Sections were mounted in ProLong Antifade Mountant (ThermoFisher). For Figures S2 and S4, stained sections were unmounted and restained with TO-PRO-3 to visualize nuclei. Each staining was replicated on the indicated number of mice per condition. Images were acquired on a spectral confocal microscope (Leica SPE) with a ×20 dry objective (N.A. 0.75). Low-magnification images were acquired with ×10 dry or a ×5 dry (N.A. 0.15) objective.

#### Image analysis

Images (×20) of RFP positive cells were uploaded to FIJI (ImageJ 1.5 g). The freehand selection tool was used to trace a region of interest (ROI) on electroporated and non-electroporated cells in the same Z section and a mean gray value measured to quantify the staining intensity of phospho-4EBP and normalized. Ratios of staining in electroporated and non-electroporated cells were compared for RFP positive cells in *Tsc2*^*w/w*^ and *Tsc2*^*mut/mut*^ conditions to account for immunohistochemical variation. All values were normalized to the average intensity of the ratio of electroporated divided by non-electroporated cells in *Tsc2*^*w/w*^ conditions.

For lesion analysis, lesions were defined in images (×20) as containing approximately eight or more overlapping RFP positive cells present within the striatum and in an individual Z section. RFP+ cells were counted in the lesion and assessed for either Sox2, p4EBP, DCX, GS, or NeuN on a per cell basis. Circularity and area were measured in maximum intensity projections. The area of the lesions was then circumscribed using the freehand selection tool and the area and circularity quantified. Hamartomas were considered Type 1 when circularity values were 0.269–0.531 and Type 2 when circularity values were 0.024–0.23.

Images (×20) were used to measure dendrite morphology. Images were uploaded as described above and simple neurite tracer plug-in was used to trace dendrite processes of RFP positive cells. Sholl analysis was performed at 10 μm intervals to quantify dendrite arborization using the Sholl plug-in. The total number of dendritic crossings were calculated by taking the sum of crossings at 10 μm intervals for each traced neuron and averaging the total number of crossings per neuron in each condition. The freehand selection tool was also used to trace the soma of neurons to measure cell size in *Tsc2*^*w/w*^ and *Tsc2*^*mut/mut*^ conditions.

Measurements were made on original image files without enhancements. Figures were created by converting LIF files into TIFF format images using FIJI. Images were uploaded into Photoshop (Adobe, version 2022). Merged images containing multiple fluorophores were subject to brightness and contrast changes and RBG levels were optimized. Changes were applied across the entirety of each image. For individual-colored images, the RBG images were duplicated, and individual colors were removed. To make black and white images, the RFP channel was converted to gray scale and inverted.

#### Total RNA isolation

Trizol was added to NSCs grown on 100 mm plates. NSC lysate was removed, triturated, and placed on ice in 1.5 mL Eppendorf tubes. Chloroform was added to tubes after freezing at −80°C and vigorously shaken by hand for 15 s. Samples were incubated for 5 min at room temperature and then centrifuged at 12,000 × g at 4°C. The upper aqueous phase was placed in a fresh tube and protein was subject to chloroform methanol precipitation for Western blot analysis. The aqueous phase was used to precipitate RNA by adding isopropanol and incubated overnight at −20°C. Samples were centrifuged for 10 min at 12,000 × g at 4°C. Supernatant was removed and pellets were washed with 75% ethanol. Samples were spun at 7,500 × g for 5 min at 4°C. Supernatant was removed and pellet air dried and resuspended in dH_2_O. Samples were incubated with DNase I and buffer RDD. Samples were then subject to Qiagen MinElute Column clean up on an RNeasy MinElute spin column. Briefly, buffer RLT is added to samples and mixed followed by addition of 100% ethanol. Samples were mixed and transferred to RNeasy MinElute spin column in a collection tube. Samples were centrifuged for 15 s at 8,000 x g twice. Columns were placed into a fresh 2 mL collection tube and 80% ethanol is added to MinElute column. Samples are centrifuged at 8,000 × g for 2 min and spin column is placed into a new tube. Samples were subject to an additional centrifugation and RNA is eluted in RNase free water and subject to quantitative and qualitative analyses.

#### Polyribosome profiling

Polyribosome profiling was performed similar to as previously described.^106^ Primary NSC cultures were passaged once prior to isolations. 100 mg/mL (66.6uL) cycloheximide (CHX, VWR, #94271) was added to each dish and incubated at 37°C for 15 min. The cells were dissociated in 500 mL lysis buffer (0.25 M sucrose, 50 mM Tris/HCl (pH7.5, Sigma Aldrich, #93363), 5 mM MgCl_2_ (Sigma Aldrich, #M1028), 25 mM KCl (Sigma Aldrich, #P9333), 200 μg/mL CHX (VWR, #94271), 1X HALT protease inhibitor (Thermo Scientific, #87785), 1 mM dithiothreitol (DTT, Enzo, #ALX-280-001-G005), 100 U/mL recombinant RNAsin ribonuclease inhibitor (Promega, #N2511), scraped into a 1.5 mL microcentrifuge tube, and incubated for 15 min on ice. Lysates were homogenized five times with a Dounce homogenizer. 50 μL was removed and 150 mL TRIzol LS reagent (Ambion, #10296010) was added for RNA Total isolation. The remaining lysates were centrifuged for 10 min at 500 × g at 4°C. 10% sodium deoxycholate and 10% NP-40 was added, and the samples were incubated on ice for 30 min. Prepared lysates were loaded onto 10 mL continuous sucrose gradients that consisted of 17.5–50% sucrose (as well as 30 mM Tris-HCl (pH7.5, Sigma Aldrich, #93363), 30 mM MgCl_2_ (Sigma Aldrich, #M1028), 600 mM NaCl (Fisher Scientific, #BP358-10), 200 μg/mL CHX (VWR, #94271), 2 mM DTT (Enzo, #ALX-280-001-G005), and UltraPure DEPC treated H_2_O (Invitrogen, #750023). The gradients were balanced using lysis buffer and were centrifuged for 1.5 h at 280,000 × g in an SW-41 Ti swinging bucket rotor with the acceleration speed set to max and the deceleration speed set to coast. Seven 1080 μL fractions were then removed from the top of the sucrose gradients leaving 3 mL of the heaviest sucrose fractions. TRIzol LS reagent (9 mL) (Ambion, #10296010) was added and stored at −80°C overnight. The solutions were then split into two equal parts and 1.2 mL of chloroform (Sigma Aldrich, #372978) was added to each tube. The tubes were incubated for 3 min before centrifugation at 12,000 × g for 15 min at 4°C. The aqueous layer was then removed and 3 mL isopropanol (Fisher Chemical, #A416-1) and 1 μL GlycoBlue co-precipitant (Invitrogen, #AM9516) was added. The tubes were then moved to the −80°C freezer to precipitate overnight. Samples were then centrifuged for 10 min at 12,000 x g at 4°C and the supernatants discarded. Pellets were resuspended in 6 mL 75% EtOH (Sigma Aldrich, #E7023) and vortexed before centrifugation for 5 min at 7,500 × g at 4°C. The supernatants were discarded, and the pellets allowed to air dry for 8 min before resuspension in 50 μL UltraPure Distilled water (Invitrogen, #10977-015). The samples were incubated for 15 min at 57°C in a water bath before being recombined into one polysomal RNA fraction each. Chloroform (Sigma Aldrich, #372978) was added to each cell lysate that was collected during polysomal profiling as a total RNA fraction. The tubes were incubated for 3 min before centrifugation at 12,000 × g for 15 min at 4C. The aqueous layer was then removed and 100 μL isopropanol (Fisher Chemical, #A416-1) and 1 μL GlycoBlue co-precipitant (Invitrogen, #AM9516) was added. The tubes were then moved to the −80°C freezer to precipitate overnight. Samples were centrifuged for 10 min at 12,000 × g at 4°C and the supernatants discarded. Pellets were resuspended in 200 μL 75% EtOH (Sigma Aldrich, #E7023) and vortexed before centrifugation for 5 min at 7,500 × g at 4°C. The supernatants were discarded, and the pellets allowed to air dry for 8 min before resuspension in 50 μL UltraPure Distilled water (Invitrogen, #10977-015). The samples were incubated for 15 min at 57°C in a water bath before library prep.

#### RNA sequencing and bioinformatics

Libraries were constructed using the NEBNext Ultra II RNA Library Prep Kit for Illumina (New England Biolabs Inc., #E7770S) and the NEBNext Poly(A) mRNA Magnetic Isolation Module (New England Biolabs Inc., #E7490S). These libraries were dual-indexed using the NEBNext Multiplex Oligos for Illumina (Dual Index Primers Set 1) (New England Biolabs Inc., #E7600S). The libraries were quantified and qualified using Qubit (Thermo-Fischer, #Q32852) and TapeStation (Agilent, # 5067–5579). Pooled libraries were sequenced using 150 bp paired-end reads on a NovaSeq 6000 SP v1.5 (300 cycles) flow cell (Illumina, #20028400). Reads were trimmed for adapter sequences using FastQ Toolkit (v. 2.2.5) and aligned to UCSC mm10 using RNA-SEQ Alignment (STAR, v. 2.6.1A) on BaseSpace (Illumina). Aligned sequences were further analyzed on BaseSpace (llumina) using RNA-SEQ Differential Expression (v. 1.0.1 which utilizes DESEQ2).^107^ Differentially enriched mRNAs were plotted by Pheatmap (R studios R package version 1.0.12. https://CRAN.R-project.org/package=pheatmap).^100^ Gene ontology analysis was performed by implementing GeneSCF^97^^,^^98^ and gene networks were identified by Cytoscape 3.60 using Genemania.^99^ Transcript and translatome ratio histograms and scatterplots were generated using tRanslatome (R package version 1.38.0).^105^

#### FACS and Single Nuclei RNA sequencing

Postnatal day 90 mice were sacrificed and V-SVZ micro-dissected as described for primary cell culture experiments. Tissue was dissected in ice-cold Neurobasal A (Gibco, #10888022) medium. 100 μL 0.25% Trypsin (Gibco, #25200072) was added and incubated for 7 min at 37°C before the tissue was dissociated using three Pasteur pipettes having progressively decreasing bore sizes. The solution was incubated for 1 min at 37°C and triturated again before being passed through a cell strainer. Samples were spun down at 300 × g for 5 min and the pellets resuspended in lysis buffer (0.25 M sucrose, 50 mM Tris/HCl (pH7.5, Sigma Aldrich, #93363), 5 mM MgCl2 (Sigma Aldrich, #M1028), 25 mM KCl (Sigma Aldrich, #P9333), 200 μg/mL cyclohexamide (VWR, #94271), 1X HALT protease inhibitor (Thermo Scientific, #87785), 1 mM dithiothreitol (DTT, Enzo, #ALX-280-001-G005), 100 U/mL recombinant RNAsin ribonuclease inhibitor (Promega, #N2511) and incubated for 15 min on ice. Lysates were homogenized five times with a Dounce homogenizer. 50 μL was removed and 150 μL TRIzol LS reagent (Ambion, #10296010) was added for RNA Total isolation. The remaining sample containing nuclei were centrifuged for 10 min at 500 × g at 4°C. The pellets were then kept at −80°C until FACs sorting. Nuclei isolated from dissected V-SVZs were resuspended in 400 μL cold Nuclei Suspension Buffer containing 1% BSA, 0.2 U/μL RNase inhibitor (RNasin Plus, Promega) in 1X PBS. The nuclei suspension was loaded on to a Bio-Rad S3e Cell Sorter and 100k tdtomato positive nuclei were sorted into chilled Nuclei Suspension Buffer. Nuclei were gated based on cell events (excluding doublets), expected fluorescence level from pilot experiments, and fluorescence microscopy verification. Samples were sorted at a rate of about 100–200 events per second based on fluorescence detection using the 586/25 nm filter. An aliquot of pre- and post- FACS nuclei were taken to confirm nuclei quality by microscopy. Nuclei samples were transported on ice until gel-bead-in-emulsion (GEM) generation. GEM generation was performed using Chromium Next GEM Chip G Single Cell Kit (10x Genomics, #1000127). GEMs were created using a 10X Genomics Chromium controller (10X Genomics, #1000204). Libraries were made after GEM generation in accordance with 10X Genomics Single Cell 3′ kit v3.1 (10× Genomics, #1000269) according to the manufacturer’s guidelines and Chromium Next GEM Single Cell 3′ v. 3.1 (Dual Index) protocol. The libraries were quantified and qualified using Qubit 4 Fluorometer (Thermo Fisher Scientific, #Q33238, 1X dsDNA, high sensitivity, 500 rxns (Thermo Fisher Scientific, #Q33231) and TapeStation 4150 (Aglient, #G2992AA, High Sensitivity D5000 ScreenTape Aglient, #5067–5592, High Sensitivity D5000 Ladder Agilent, #5067–5594. Final libraries were pooled on a NovaSeq 6000 SP v1.5 (300 cycles) flow cell (Illumina, #20028400) according to manufacturer’s instructions.

#### Single Nuclei RNA sequencing bioinformatics

BCL files from the sequencer were demultiplexed and converted to FASTQ files using the *mkfastq* command and default parameters within the Cell Ranger 7.0.1 pipeline. FASTQ files were preprocessed for quality and length and aligned to *Mus musculus* reference genome GRCm38 (mm10) using the *counts* command with *-force-cells* parameter set to 60000 to account for spurious cell identity in one of the samples. This number was derived by calculating the median and rounding to the nearest 10000 from the unaffected samples part of the sequenced set. The output from the *counts* command was exported into R for quality control, multi-sample integration, cell-type clustering, cell-type marker identification and differential expression within each cluster using the Seurat v4 single cell analysis platform^108^ and previously described methods.^109^^,^^110^ Differential expression lists were curated by removing mitochondrial encoded transcripts. The bash and R code for Cell Ranger and Seurat pipeline, respectively can be found here: https://github.com/vshanka23/tsc_scrna_feliciano.

#### Western blot

Lysates from NSCs subject to Trizol RNA isolation were further processed by chloroform methanol isolations. Proteins were placed into Laemmli buffer and heated to 95°C for 5 min. Samples were resolved by electrophoresis on 10% polyacrylamide precast mini-Protean gels and transferred to polyvinylidene difluoride (PVDF) membranes. Blots were cut to probe upper vs. lower parts of the same blot for different proteins. Blots were rinsed in Tris-buffered saline (TBS-T, 0.1% Tween 20) for 5 min at room temperature and subsequently blocked in 5% weight/volume blotting grade block in TBS-T for 1 h at room temperature. Samples were incubated for 1 h at room temperature or overnight at 4°C with the following antibodies from Cell Signaling Technology at a 1:1,000 dilution: Tuberin (D93F12, Cat#4308), ATF4 (D4B8, Cat# 11815), Actin (13E5, Cat# 4970), phospho-RPS6 (D68F8, Cat# 5364), RPL26 (D8F6, Cat#5400), RPS3 (D50G7, Cat# 9538), and RPS6 (5G10, Cat# 2217). Blots were washed three times each for 10 min in TBS-T, samples were incubated for 1 h at room temperature with donkey or goat anti-rabbit antibodies in blocking buffer and washed for 15 min in TBS-T and visualized using a Bio-Rad Chemidoc MP imaging system. PVDF membranes were stripped for at room temperature using Restore Western Blot Stripping Buffer according to manufacturer’s recommendations (#21059, Thermo Fisher Scientific).

### Quantification and statistical analysis

Data were graphed and analyzed with GraphPad Prism software (Version 8.2.0, GraphPad Software Inc.). Statistical significance was determined by Student’s *t* test where indicated (p4EBP quantification [Figure 1H p < 0.0001, L p < 0.0001], hamartoma circularity [Figure 1M, p < 0.0001], hamartoma size [Figure 1N, p < 0.001], sholl analysis, [Figure 1T, p < 0.0001], number of neurons [Figure S3A, p < 0.001, B, p < 0.001], soma size [Figure S3E, p < 0.0001], Western blot [Figure S4E, p < 0.05], NSC growth in culture [Figure S6C, p < 0.05]). One-way analysis of variance (ANOVA) with Tukey’s multiple comparisons test was performed for Figure 4J, ∗ = p < 0.05, ∗∗ = p < 0.01, ∗∗∗ = p < 0.001). p values less than 0.05 were considered significant unless otherwise noted. No methods were used to determine whether the data met assumptions of the statistical approach. Quantification and statistics for single cell RNA sequencing (Figures 4A and 4B, N = 4 mice per genotype, Seurat), polyribosome profiling (Figures 3A–3D, N = 4 cultures per genotype, DeSeq2 and tRanslatome, p adj. <0.00001), bulk RNA sequencing (Figures 2A and 2B, N = 3 cultures per genotype, DeSeq2, p adj. <0.00001), and GO term analysis (Figure 2E, N = 4 mice per genotype; Figures 3E and 3F, N = 4 mice per genotype, p < 0.05) was determined using the indicated programs for bioinformatic analyses. All experiments were performed on 6–17 mice per condition per time point. N (number of mice) and n (number of cells or hamartomas) are listed where applicable. Error bars are reported as standard error mean.

## Supplemental information

Document S1. Figures S1–S9

Table S1. Top 10 Single Nuclei RNA Sequencing Cluster Markers, related to Figure 4A

Table S2. Single Nuclei RNA Sequencing Differentially Expressed Transcripts, related to Figure 4C-D

## References

[1] Lim D.A., Alvarez-Buylla A. (2016). The adult ventricular–subventricular zone (V-SVZ) and olfactory bulb (OB) neurogenesis. Cold Spring Harb. Perspect. Biol..

[2] Bonaguidi M.A., Stadel R.P., Berg D.A., Sun J., Ming G.L., Song H. (2016). Diversity of neural precursors in the adult mammalian brain. Cold Spring Harb. Perspect. Biol..

[3] Beckervordersandforth R., Tripathi P., Ninkovic J., Bayam E., Lepier A., Stempfhuber B., Kirchhoff F., Hirrlinger J., Haslinger A., Lie D.C. (2010). In vivo fate mapping and expression analysis reveals molecular hallmarks of prospectively isolated adult neural stem cells. Cell Stem Cell.

[4] Young K.M., Fogarty M., Kessaris N., Richardson W.D. (2007). Subventricular zone stem cells are heterogeneous with respect to their embryonic origins and neurogenic fates in the adult olfactory bulb. J. Neurosci..

[5] Xie X.P., Laks D.R., Sun D., Poran A., Laughney A.M., Wang Z., Sam J., Belenguer G., Fariñas I., Elemento O. (2020). High-resolution mouse subventricular zone stem-cell niche transcriptome reveals features of lineage, anatomy, and aging. Proc. Natl. Acad. Sci. USA.

[6] Cebrian-Silla A., Nascimento M.A., Redmond S.A., Mansky B., Wu D., Obernier K., Romero Rodriguez R., Gonzalez-Granero S., García-Verdugo J.M., Lim D.A., Álvarez-Buylla A. (2021). Single-cell analysis of the ventricular-subventricular zone reveals signatures of dorsal and ventral adult neurogenic lineages. Elife.

[7] Basak O., Krieger T.G., Muraro M.J., Wiebrands K., Stange D.E., Frias-Aldeguer J., Rivron N.C., van de Wetering M., van Es J.H., van Oudenaarden A. (2018). Troy+ brain stem cells cycle through quiescence and regulate their number by sensing niche occupancy. Proc. Natl. Acad. Sci. USA.

[8] Choe Y., Pleasure S.J., Mira H. (2016). Control of adult neurogenesis by short-range morphogenic-signaling molecules. Cold Spring Harb. Perspect. Biol..

[9] Wiebe S., Nagpal A., Sonenberg N. (2020). Dysregulated translational control in brain disorders: from genes to behavior. Curr. Opin. Genet. Dev..

[10] Lipton J.O., Sahin M. (2014). The Neurology of mTOR. Neuron.

[11] Crino P.B. (2015). mTOR signaling in epilepsy: Insights from malformations of cortical development. Cold Spring Harb. Perspect. Méd..

[12] Northrup H., Aronow M.E., Bebin E.M., Bissler J., Darling T.N., de Vries P.J., Frost M.D., Fuchs Z., Gosnell E.S., Gupta N. (2021). Updated International Tuberous Sclerosis Complex Diagnostic Criteria and Surveillance and Management Recommendations. Pediatr. Neurol..

[13] European Chromosome 16 Tuberous Sclerosis Consortium (1993). Identification and characterization of the tuberous sclerosis gene on chromosome 16. Cell.

[14] Van Slegtenhorst M., De Hoogt R., Hermans C., Nellist M., Janssen B., Verhoef S., Lindhout D., Van Den Ouweland A., Halley D., Young J. (1997). Identification of the tuberous sclerosis gene TSC1 on chromosome 9q34. Science.

[15] Inoki K., Zhu T., Guan K.L. (2003). TSC2 Mediates Cellular Energy Response to Control Cell Growth and Survival. Cell.

[16] Tee A.R., Manning B.D., Roux P.P., Cantley L.C., Blenis J. (2003). Tuberous Sclerosis Complex gene products, Tuberin and Hamartin, control mTOR signaling by acting as a GTPase-activating protein complex toward Rheb. Curr. Biol..

[17] Garami A., Zwartkruis F.J.T., Nobukuni T., Joaquin M., Roccio M., Stocker H., Kozma S.C., Hafen E., Bos J.L., Thomas G. (2003). Insulin activation of Rheb, a mediator of mTOR/S6K/4E-BP signaling, is inhibited by TSC1 and 2. Mol. Cell.

[18] Zhang Y., Gao X., Saucedo L.J., Ru B., Edgar B.A., Pan D. (2003). Rheb is a direct target of the tuberous sclerosis tumour suppressor proteins. Nat. Cell Biol..

[19] Feliciano D.M. (2020). The Neurodevelopmental Pathogenesis of Tuberous Sclerosis Complex (TSC). Front. Neuroanat..

[20] de Vries P.J., Belousova E., Benedik M.P., Carter T., Cottin V., Curatolo P., Dahlin M., D’Amato L., d’Augères G.B., Ferreira J.C. (2018). TSC-associated neuropsychiatric disorders (TAND): findings from the TOSCA natural history study. Orphanet J. Rare Dis..

[21] Kingswood J.C., D’Augères G.B., Belousova E., Ferreira J.C., Carter T., Castellana R., Cottin V., Curatolo P., Dahlin M., De Vries P.J. (2017). TuberOus SClerosis registry to increase disease Awareness (TOSCA) - Baseline data on 2093 patients. Orphanet J. Rare Dis..

[22] Nabbout R., Belousova E., Benedik M.P., Carter T., Cottin V., Curatolo P., Dahlin M., D'Amato L., d'Augères G.B., de Vries P.J. (2019). Epilepsy in tuberous sclerosis complex: Findings from the TOSCA Study. Epilepsia Open.

[23] Lachhwani D.K., Pestana E., Gupta A., Kotagal P., Bingaman W., Wyllie E. (2005). Identification of candidates for epilepsy surgery in patients with tuberous sclerosis. Neurology.

[24] Chugani D.C., Chugani H.T., Muzik O., Shah J.R., Shah A.K., Canady A., Mangner T.J., Chakraborty P.K. (1998). Imaging epileptogenic tubers in children with tuberous sclerosis complex using α-[11C]methyl-L-tryptophan positron emission tomography. Ann. Neurol..

[25] Fallah A., Rodgers S.D., Weil A.G., Vadera S., Mansouri A., Connolly M.B., Major P., Ma T., Devinsky O., Weiner H.L. (2015). Resective Epilepsy Surgery for Tuberous Sclerosis in Children: Determining Predictors of Seizure Outcomes in a Multicenter Retrospective Cohort Study. Neurosurgery.

[26] Mohamed A.R., Bailey C.A., Freeman J.L., Maixner W., Jackson G.D., Harvey A.S. (2012). Intrinsic epileptogenicity of cortical tubers revealed by intracranial EEG monitoring. Neurology.

[27] Doherty C., Goh S., Young Poussaint T., Erdag N., Thiele E.A. (2005). Prognostic significance of tuber count and location in tuberous sclerosis complex. J. Child Neurol..

[28] Canevini M.P., Kotulska-Jozwiak K., Curatolo P., La Briola F., Peron A., Słowińska M., Strzelecka J., Vignoli A., Jóźwiak S. (2018). Current concepts on epilepsy management in tuberous sclerosis complex. Am J Med Genet C Semin Med Genet..

[29] Nabbout R., Belousova E., Benedik M.P., Carter T., Cottin V., Curatolo P., Dahlin M., D’Amato L., Beaure d'Augères G., de Vries P.J. (2021). Historical Patterns of Diagnosis, Treatments, and Outcome of Epilepsy Associated With Tuberous Sclerosis Complex: Results From TOSCA Registry. Front. Neurol..

[30] Fallah A., Guyatt G.H., Snead O.C., Ebrahim S., Ibrahim G.M., Mansouri A., Reddy D., Walter S.D., Kulkarni A.V., Bhandari M. (2013). Predictors of Seizure Outcomes in Children with Tuberous Sclerosis Complex and Intractable Epilepsy Undergoing Resective Epilepsy Surgery: An Individual Participant Data Meta-Analysis. PLoS One.

[31] Mühlebner A., Van Scheppingen J., Hulshof H.M., Scholl T., Iyer A.M., Anink J.J., Nellist M.D., Jansen F.E., Spliet W.G.M., Krsek P. (2016). Novel histopathological patterns in cortical tubers of epilepsy surgery patients with tuberous sclerosis complex. PLoS One.

[32] Rakic P. (2003). Elusive Radial Glial Cells: Historical and Evolutionary Perspective. Glia.

[33] Noctor S.C., Martínez-Cerdeño V., Ivic L., Kriegstein A.R. (2004). Cortical neurons arise in symmetric and asymmetric division zones and migrate through specific phases. Nat. Neurosci..

[34] Meikle L., Talos D.M., Onda H., Pollizzi K., Rotenberg A., Sahin M., Jensen F.E., Kwiatkowski D.J. (2007). A mouse model of tuberous sclerosis: Neuronal loss of Tsc1 causes dysplastic and ectopic neurons, reduced myelination, seizure activity, and limited survival. J. Neurosci..

[35] Way S.W., Mckenna J., Mietzsch U., Reith R.M., Wu H.C.J., Gambello M.J. (2009). Loss of Tsc2 in radial glia models the brain pathology of tuberous sclerosis complex in the mouse. Hum. Mol. Genet..

[36] Moon U.Y., Park J.Y., Park R., Cho J.Y., Hughes L.J., McKenna J., Goetzl L., Cho S.H., Crino P.B., Gambello M.J., Kim S. (2015). Impaired Reelin-Dab1 signaling contributes to neuronal migration deficits of tuberous sclerosis complex. Cell Rep..

[37] Goto J., Talos D.M., Klein P., Qin W., Chekaluk Y.I., Anderl S., Malinowska I.A., Di Nardo A., Bronson R.T., Chan J.A. (2011). Regulable neural progenitor-specific Tsc1 loss yields giant cells with organellar dysfunction in a model of tuberous sclerosis complex. Proc. Natl. Acad. Sci. USA.

[38] Magri L., Cambiaghi M., Cominelli M., Alfaro-Cervello C., Cursi M., Pala M., Bulfone A., Garcìa-Verdugo J.M., Leocani L., Minicucci F. (2011). Sustained activation of mTOR pathway in embryonic neural stem cells leads to development of tuberous sclerosis complex-associated lesions. Cell Stem Cell.

[39] Feliciano D.M., Su T., Lopez J., Platel J.C., Bordey A. (2011). Single-cell Tsc1 knockout during corticogenesis generates tuber-like lesions and reduces seizure threshold in mice. J. Clin. Invest..

[40] Cox R.L., de Anda F.C., Mangoubi T., Yoshii A. (2018). Multiple critical periods for rapamycin treatment to correct structural defects in Tsc-1-suppressed brain. Front. Mol. Neurosci..

[41] Lim J.S., Gopalappa R., Kim S.H., Ramakrishna S., Lee M., Kim W.I., Park S.M., Lee J., Oh J.H., Kim H.D. (2017). Somatic Mutations in TSC1 and TSC2 Cause Focal Cortical Dysplasia. Am. J. Hum. Genet..

[42] Malik R., Pai E.L.L., Rubin A.N., Stafford A.M., Angara K., Minasi P., Rubenstein J.L., Sohal V.S., Vogt D. (2019). Tsc1 represses parvalbumin expression and fast-spiking properties in somatostatin lineage cortical interneurons. Nat. Commun..

[43] Fu C., Cawthon B., Clinkscales W., Bruce A., Winzenburger P., Ess K.C. (2012). GABAergic interneuron development and function is modulated by the Tsc1 gene. Cerebr. Cortex.

[44] Marcotte L., Aronica E., Baybis M., Crino P.B. (2012). Cytoarchitectural alterations are widespread in cerebral cortex in tuberous sclerosis complex. Acta Neuropathol..

[45] Cotter J.A. (2020). An update on the central nervous system manifestations of tuberous sclerosis complex. Acta Neuropathol..

[46] Jansen A.C., Belousova E., Benedik M.P., Carter T., Cottin V., Curatolo P., D’Amato L., Beaure d'Augères G., De Vries P.J., Ferreira J.C. (2019). Newly diagnosed and growing subependymal giant cell astrocytoma in adults with tuberous sclerosis complex: Results from the International TOSCA Study. Front. Neurol..

[47] Zordan P., Cominelli M., Cascino F., Tratta E., Poliani P.L., Galli R. (2018). Tuberous sclerosis complex-associated CNS abnormalities depend on hyperactivation of mTORC1 and Akt. J. Clin. Invest..

[48] Gelot A.B., Represa A. (2020). Progression of Fetal Brain Lesions in Tuberous Sclerosis Complex. Front. Neurosci..

[49] Sanai H., Tramontin A.D., Quiñones-Hinojosa A., Barbaro N.M., Gupta H., Kunwar S., Lawton M.T., McDermott M.W., Parsa A.T., Verdugo J.M.G. (2004). Unique astrocyte ribbon in adult human brain contains neural stem cells but lacks chain migration. Nature.

[50] Quiñones-Hinojosa A., Sanai N., Soriano-Navarro M., Gonzalez-Perez O., Mirzadeh Z., Gil-Perotin S., Romero-Rodriguez R., Berger M.S., Garcia-Verdugo J.M., Alvarez-Buylla A. (2006). Cellular composition and cytoarchitecture of the adult human subventricular zone: A niche of neural stem cells. J. Comp. Neurol..

[51] Sanai N., Nguyen T., Ihrie R.A., Mirzadeh Z., Tsai H.H., Wong M., Gupta N., Berger M.S., Huang E., Garcia-Verdugo J.M. (2011). Corridors of migrating neurons in the human brain and their decline during infancy. Nature.

[52] Eichmüller O.L., Corsini N.S., Vértesy Á., Morassut I., Scholl T., Gruber V.E., Peer A.M., Chu J., Novatchkova M., Hainfellner J.A. (2022). Amplification of human interneuron progenitors promotes brain tumors and neurological defects. Science.

[53] Kwiatkowski D.J., Manning B.D. (2014). Molecular basis of giant cells in tuberous sclerosis complex. N. Engl. J. Med..

[54] Lopes M.B.S., Altermatt H.J., Scheithauer B.W., Shepherd C.W., VandenBerg S.R. (1996). Immunohistochemical characterization of subependymal giant cell astrocytomas. Acta Neuropathol..

[55] Phi J.H., Park S.H., Chae J.H., Hong K.H., Park S.S., Kang J.H., Jun J.K., Cho B.K., Wang K.C., Kim S.K. (2008). Congenital subependymal giant cell astrocytoma: Clinical considerations and expression of radial glial cell markers in giant cells. Child’s Nerv. Off. Syst..

[56] Tyburczy M.E., Kotulska K., Pokarowski P., Mieczkowski J., Kucharska J., Grajkowska W., Roszkowski M., Jozwiak S., Kaminska B. (2010). Novel proteins regulated by mTOR in subependymal giant cell astrocytomas of patients with tuberous sclerosis complex and new therapeutic implications. Am. J. Pathol..

[57] Martin K.R., Zhou W., Bowman M.J., Shih J., Au K.S., Dittenhafer-Reed K.E., Sisson K.A., Koeman J., Weisenberger D.J., Cottingham S.L. (2017). The genomic landscape of tuberous sclerosis complex. Nat. Commun. Now..

[58] Bongaarts A., Van Scheppingen J., Korotkov A., Mijnsbergen C., Anink J.J., Jansen F.E., Spliet W.G.M., Den Dunnen W.F.A., Gruber V.E., Scholl T. (2020). The coding and non-coding transcriptional landscape of subependymal giant cell astrocytomas. Brain.

[59] Bongaarts A., Giannikou K., Reinten R.J., Anink J.J., Mills J.D., Jansen F.E., Spliet G.M.W., den Dunnen W.F.A., Coras R., Blümcke I. (2017). Subependymal giant cell astrocytomas in Tuberous Sclerosis Complex have consistent TSC1/TSC2 biallelic inactivation, and no BRAF mutations. Oncotarget.

[60] Zhou J., Shrikhande G., Xu J., Mckay R.M., Burns D.K., Johnson J.E., Parada L.F. (2011). Tsc1 mutant neural stem/progenitor cells exhibit migration deficits and give rise to subependymal lesions in the lateral ventricle. Genes Dev..

[61] Feliciano D.M., Quon J.L., Su T., Taylor M.M., Bordey A. (2012). Postnatal neurogenesis generates heterotopias, olfactory micronodules and cortical infiltration following single-cell TSC1 deletion. Hum. Mol. Genet..

[62] Zucco A.J., Pozzo V.D., Afinogenova A., Hart R.P., Devinsky O., D’Arcangelo G. (2018). Neural progenitors derived from Tuberous Sclerosis Complex patients exhibit attenuated PI3K/AKT signaling and delayed neuronal differentiation. Mol. Cell. Neurosci..

[63] Blair J.D., Hockemeyer D., Bateup H.S. (2018). Genetically engineered human cortical spheroid models of tuberous sclerosis. Nat. Med..

[64] Uhlmann E.J., Wong M., Baldwin R.L., Bajenaru M.L., Onda H., Kwiatkowski D.J., Yamada K., Gutmann D.H. (2002). Astrocyte-specific TSC1 conditional knockout mice exhibit abnormal neuronal organization and seizures. Ann. Neurol..

[65] Zeng L.H., Rensing N.R., Zhang B., Gutmann D.H., Gambello M.J., Wong M. (2011). Tsc2 gene inactivation causes a more severe epilepsy phenotype than Tsc1 inactivation in a mouse model of Tuberous Sclerosis Complex. Hum. Mol. Genet..

[66] Zou J., Zhang B., Gutmann D.H., Wong M. (2017). Postnatal reduction of tuberous sclerosis complex 1 expression in astrocytes and neurons causes seizures in an age-dependent manner. Epilepsia.

[67] Jansen A.C., Belousova E., Benedik M.P., Carter T., Cottin V., Curatolo P., Dahlin M., D’Amato L., d'Augères G.B., De Vries P.J. (2019). Clinical characteristics of subependymal giant cell astrocytoma in tuberous sclerosis complex. Front. Neurol..

[68] Rushing G.V., Brockman A.A., Bollig M.K., Leelatian N., Mobley B.C., Irish J.M., Ess K.C., Fu C., Ihrie R.A. (2019). Location-dependent maintenance of intrinsic susceptibility to mTORC1-driven tumorigenesis. Life Sci. Alliance.

[69] García-González D., Dumitru I., Zuccotti A., Yen T.Y., Herranz-Pérez V., Tan L.L., Neitz A., García-Verdugo J.M., Kuner R., Alfonso J. (2021). Neurogenesis of medium spiny neurons in the nucleus accumbens continues into adulthood and is enhanced by pathological pain. Mol. Psychiatr..

[70] Riley V.A., Holmberg J.C., Sokolov A.M., Feliciano D.M. (2022). Tsc2 shapes olfactory bulb granule cell molecular and morphological characteristics. Front. Mol. Neurosci..

[71] Niu W., Zang T., Zou Y., Fang S., Smith D.K., Bachoo R., Zhang C.L. (2013). In vivo reprogramming of astrocytes to neuroblasts in the adult brain. Nat. Cell Biol..

[72] Niu W., Zang T., Smith D.K., Vue T.Y., Zou Y., Bachoo R., Johnson J.E., Zhang C.L. (2015). SOX2 reprograms resident astrocytes into neural progenitors in the adult brain. Stem Cell Rep..

[73] Wu Z., Parry M., Hou X.Y., Liu M.H., Wang H., Cain R., Pei Z.F., Chen Y.C., Guo Z.Y., Abhijeet S., Chen G. (2020). Gene therapy conversion of striatal astrocytes into GABAergic neurons in mouse models of Huntington’s disease. Nat. Commun..

[74] Torper O., Ottosson D., Pereira M., Lau S., Cardoso T., Grealish S., Parmar M. (2015). InVivo Reprogramming of Striatal NG2 Glia into Functional Neurons that Integrate into Local Host Circuitry. Cell Rep..

[75] Bongaarts A., Mijnsbergen C., Anink J.J., Jansen F.E., Spliet W.G.M., den Dunnen W.F.A., Coras R., Blümcke I., Paulus W., Gruber V.E. (2021). Distinct DNA Methylation Patterns of Subependymal Giant Cell Astrocytomas in Tuberous Sclerosis Complex. Cell. Mol. Neurobiol..

[76] Giannikou K., Zhu Z., Kim J., Winden K.D., Tyburczy M.E., Marron D., Parker J.S., Hebert Z., Bongaarts A., Taing L. (2021). Subependymal giant cell astrocytomas are characterized by mTORC1 hyperactivation, a very low somatic mutation rate, and a unique gene expression profile. Mod. Pathol..

[77] Ercan E., Han J.M., Di Nardo A., Winden K., Han M.J., Hoyo L., Saffari A., Leask A., Geschwind D.H., Sahin M. (2017). Neuronal CTGF/CCN2 negatively regulates myelination in a mouse model of tuberous sclerosis complex. J. Exp. Med..

[78] Baser A., Skabkin M., Kleber S., Dang Y., Gülcüler Balta G.S., Kalamakis G., Göpferich M., Ibañez D.C., Schefzik R., Lopez A.S. (2019). Onset of differentiation is post-transcriptionally controlled in adult neural stem cells. Nature.

[79] Paliouras G.N., Hamilton L.K., Aumont A., Joppé S.E., Barnabé-Heider F., Fernandes K.J.L. (2012). Mammalian target of rapamycin signaling is a key regulator of the transit-amplifying progenitor pool in the adult and aging forebrain. J. Neurosci..

[80] Hartman N.W., Lin T.V., Zhang L., Paquelet G.E., Feliciano D.M., Bordey A. (2013). MTORC1 Targets the Translational Repressor 4E-BP2, but Not S6 Kinase 1/2, to Regulate Neural Stem Cell Self-Renewal InVivo. Cell Rep..

[81] Blair J.D., Hockemeyer D., Doudna J.A., Bateup H.S., Floor S.N. (2017). Widespread Translational Remodeling during Human Neuronal Differentiation. Cell Rep..

[82] Onda H., Crino P.B., Zhang H., Murphey R.D., Rastelli L., Rothberg B.E.G., Kwiatkowski D.J. (2002). Tsc2 null murine neuroepithelial cells are a model for human tuber giant cells, and show activation of an mTOR pathway. Mol. Cell. Neurosci..

[83] Mietzsch U., McKenna J., Reith R.M., Way S.W., Gambello M.J. (2013). Comparative analysis of Tsc1 and Tsc2 single and double radial glial cell mutants. J. Comp. Neurol..

[84] Kassai H., Sugaya Y., Noda S., Nakao K., Maeda T., Kano M., Aiba A. (2014). Selective Activation of mTORC1 Signaling Recapitulates Microcephaly, Tuberous Sclerosis, and Neurodegenerative Diseases. Cell Rep..

[85] Alcantara Llaguno S., Sun D., Pedraza A.M., Vera E., Wang Z., Burns D.K., Parada L.F. (2019). Cell-of-origin susceptibility to glioblastoma formation declines with neural lineage restriction. Nat. Neurosci..

[86] Zhu G., Chow L.M.L., Bayazitov I.T., Tong Y., Gilbertson R.J., Zakharenko S.S., Solecki D.J., Baker S.J. (2012). Pten deletion causes mTorc1-dependent ectopic neuroblast differentiation without causing uniform migration defects. Devenir.

[87] Ridler K., Suckling J., Higgins N.J., De Vries P.J., Stephenson C.M.E., Bolton P.F., Bullmore E.T. (2007). Neuroanatomical correlates of memory deficits in tuberous sclerosis complex. Cerebr. Cortex.

[88] Arvidsson A., Collin T., Kirik D., Kokaia Z., Lindvall O. (2002). Neuronal replacement from endogenous precursors in the adult brain after stroke. Nat. Med..

[89] Ernst A., Alkass K., Bernard S., Salehpour M., Perl S., Tisdale J., Possnert G., Druid H., Frisén J. (2014). Neurogenesis in the striatum of the adult human brain. Cell.

[90] Magnusson J.P., Göritz C., Tatarishvili J., Dias D.O., Smith E.M.K., Lindvall O., Kokaia Z., Frisén J. (2014). A latent neurogenic program in astrocytes regulated by Notch signaling in the mouse. Science.

[91] Magnusson J.P., Zamboni M., Santopolo G., Mold J.E., Barrientos-Somarribas M., Talavera-Lopez C., Andersson B., Frisén J. (2020). Activation of a neural stem cell transcriptional program in parenchymal astrocytes. Elife.

[92] Ma J., Meng Y., Kwiatkowski D.J., Chen X., Peng H., Sun Q., Zha X., Wang F., Wang Y., Jing Y. (2010). Mammalian target of rapamycin regulates murine and human cell differentiation through STAT3/p63/Jagged/Notch cascade. J. Clin. Invest..

[93] Cho J.H., Patel B., Bonala S., Manne S., Zhou Y., Vadrevu S.K., Patel J., Peronaci M., Ghouse S., Henske E.P. (2017). Notch transactivates Rheb to maintain the multipotency of TSC-null cells. Nat. Commun..

[94] Matsuda T., Cepko C.L. (2007). Controlled expression of transgenes introduced by in vivo electroporation. Proc. Natl. Acad. Sci. USA.

[95] Matsuda T., Cepko C.L. (2004). Electroporation and RNA interference in the rodent retina in vivo and in vitro. Proc. Natl. Acad. Sci. USA.

[96] Schindelin J., Arganda-Carreras I., Frise E., Kaynig V., Longair M., Pietzsch T., Preibisch S., Rueden C., Saalfeld S., Schmid B. (2012). Fiji: An open-source platform for biological-image analysis. Nat. Methods.

[97] Ashburner M., Ball C.A., Blake J.A., Botstein D., Butler H., Cherry J.M., Davis A.P., Dolinski K., Dwight S.S., Eppig J.T. (2000). Gene ontology: Tool for the unification of biology. Nat. Genet..

[98] Carbon S., Douglass E., Good B.M., Unni D.R., Harris N.L., Mungall C.J., Basu S., Chisholm R.L., Dodson R.J., Hartline E. (2021). The Gene Ontology resource: Enriching a GOld mine. Nucleic Acids Res..

[99] Warde-Farley D., Donaldson S.L., Comes O., Zuberi K., Badrawi R., Chao P., Franz M., Grouios C., Kazi F., Lopes C.T. (2010). The GeneMANIA prediction server: Biological network integration for gene prioritization and predicting gene function. Nucleic Acids Res..

[100] Kolde R. (2019).

[101] Tebaldi T., Dassi E., Kostoska G., Viero G., Quattrone A. (2014). TRanslatome: An R/Bioconductor package to portray translational control. Bioinformatics.

[102] Stuart T., Butler A., Hoffman P., Hafemeister C., Papalexi E., Mauck W.M., Hao Y., Stoeckius M., Smibert P., Satija R. (2019). Seurat v3. Cell.

[103] Lagace D.C., Whitman M.C., Noonan M.A., Ables J.L., DeCarolis N.A., Arguello A.A., Donovan M.H., Fischer S.J., Farnbauch L.A., Beech R.D. (2007). Dynamic contribution of nestin-expressing stem cells to adult neurogenesis. J. Neurosci..

[104] Hernandez O., Way S., McKenna J., Gambello M.J. (2007). Generation of a conditional disruption of the Tsc2 gene. Genesis.

[105] Feliciano D.M., Lafourcade C.A., Bordey A. (2013). Neonatal subventricular zone electroporation. J. Vis. Exp..

[106] Lou W.P.K., Baser A., Klußmann S., Martin-Villalba A. (2014). In vivo interrogation of central nervous system translatome by polyribosome fractionation. J. Vis. Exp..

[107] Love M.I., Huber W., Anders S. (2014). Moderated estimation of fold change and dispersion for RNA-seq data with DESeq2. Genome Biol..

[108] Hao Y., Hao S., Andersen-Nissen E., Mauck W.M., Zheng S., Butler A., Lee M.J., Wilk A.J., Darby C., Zager M. (2021). Integrated analysis of multimodal single-cell data. Cell.

[109] Brandon M.B., Mokashi S.S., Vijay S., Hatfield J.S., Hannah R.C., Trudy F.C., Mackay R.R.H.A. (2021). The Drosophila brain on cocaine at single-cell resolution. Genome Res..

[110] Mokashi S.S., Shankar V., MacPherson R.A., Hannah R.C., Mackay T.F.C., Anholt R.R.H. (2021). Developmental Alcohol Exposure in Drosophila: Effects on Adult Phenotypes and Gene Expression in the Brain. Front. Psychiatry.

