# Supplementary material for: Tsc2 coordinates neuroprogenitor differentiation
Source: iScience. 2023 Nov 14;26(12):108442. doi: 10.1016/j.isci.2023.108442 (PMC10724693; doi:10.1016/j.isci.2023.108442)
Supplement: Document S1. Figures S1–S9 [file mmc1.pdf]

iScience, Volume *26*

Supplemental information

Tsc2 coordinates neuroprogenitor differentiation

Victoria A. Riley, Vijay Shankar, Jennie C. Holmberg, Aidan M. Sokolov, Victoria N. Neckles, Kaitlyn Williams, Rachel Lyman, Trudy F.C. Mackay, and David M. Feliciano

Supplemental information.

Tsc2 coordinates neuroprogenitor differentiation

Riley, Victoria A.[1], Shankar, Vijay[2, 3], Holmberg, Jennie C.[1], Sokolov, Aidan M.[1], Neckles, Victoria N.[1], Williams, Kaitlyn[4], Lyman, Rachel[2, 3], Mackay, Trudy F.C.[2, 3], Feliciano, David M.[1, 3, 5, *]

1. Department of Biological Sciences, Clemson University, Clemson, South Carolina, United States of America
2. Department of Biochemistry and Genetics, Clemson University, Clemson, South Carolina, United States of America
3. Center for Human Genetics, Clemson University, Greenwood, South Carolina, United States of America.
4. Clemson University Genomics and Bioinformatics Facility (CUGBF), Clemson University, Clemson South Carolina, United States of America
5. Lead Contact

*Correspondence: David M. Feliciano, Ph.D., Department of Biological Sciences, Clemson University, Clemson, SC 29634-0314, USA. Tel: 864.656.2328 FAX: 864.656.0435; E-mail: dfelici@clemson.edu

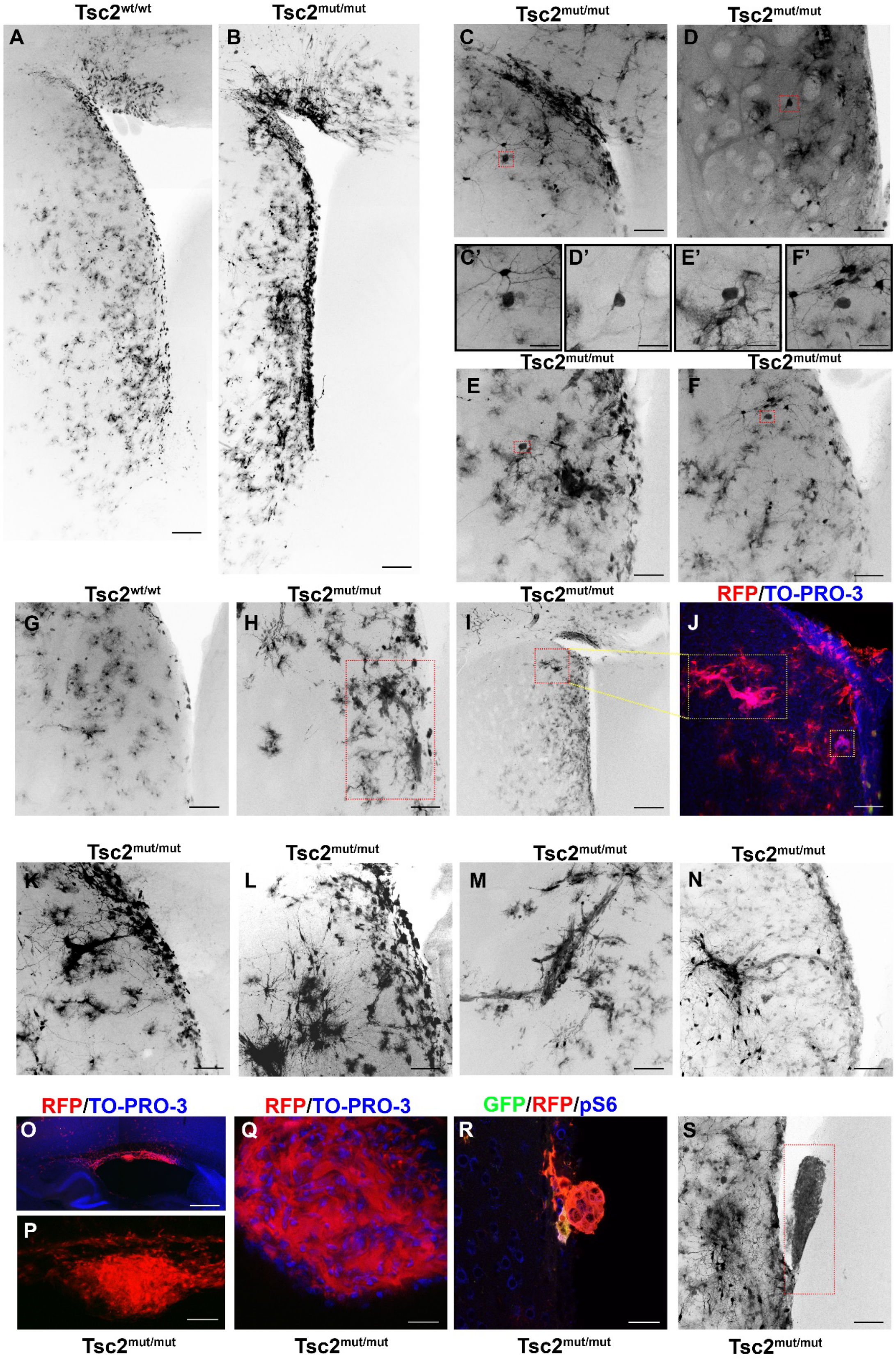

Tsc2 mutant mice develop hamartomas, related to Figure 1

Supplemental Figure 1. Images are of RFP and were converted to black and white unless otherwise noted. A) Composite image of *Tsc2*$^{wt/wt}$ cells along the V-SVZ. Note the cellular distribution within the striatum. B. Composite image of *Tsc2*$^{mut/mut}$ cells along the V-SVZ. Note the large overlapping cell clusters. C-F. 20x images of giant cells highlighted with red squares with digital zoom C'-F'. Note their distinct soma compared to ectopically

position cytomegalic neurons in lateral striatum. G) *Tsc2*$^{wt/wt}$ or (H) *Tsc2*$^{mut/mut}$ cells within the lateral striatum. I) 5x image of *Tsc2*$^{mut/mut}$ mouse with minor hamartoma highlighted. J) 20x image of the same hamartoma in (I). K-N) 20x Images of *Tsc2*$^{mut/mut}$ striatal hamartomas. O) Composite or P-S) individual examples of hamartomas that breach the walls of the lateral ventricles. A-B scale bar = 150 μm. C-H, J-N, S scale bar = 75 μm. C'-F' scale bar = 18.75 μm. I, O scale bar = 300 μm. J scale bar = 75 μm. Q, R scale bar = 23.8 μm.

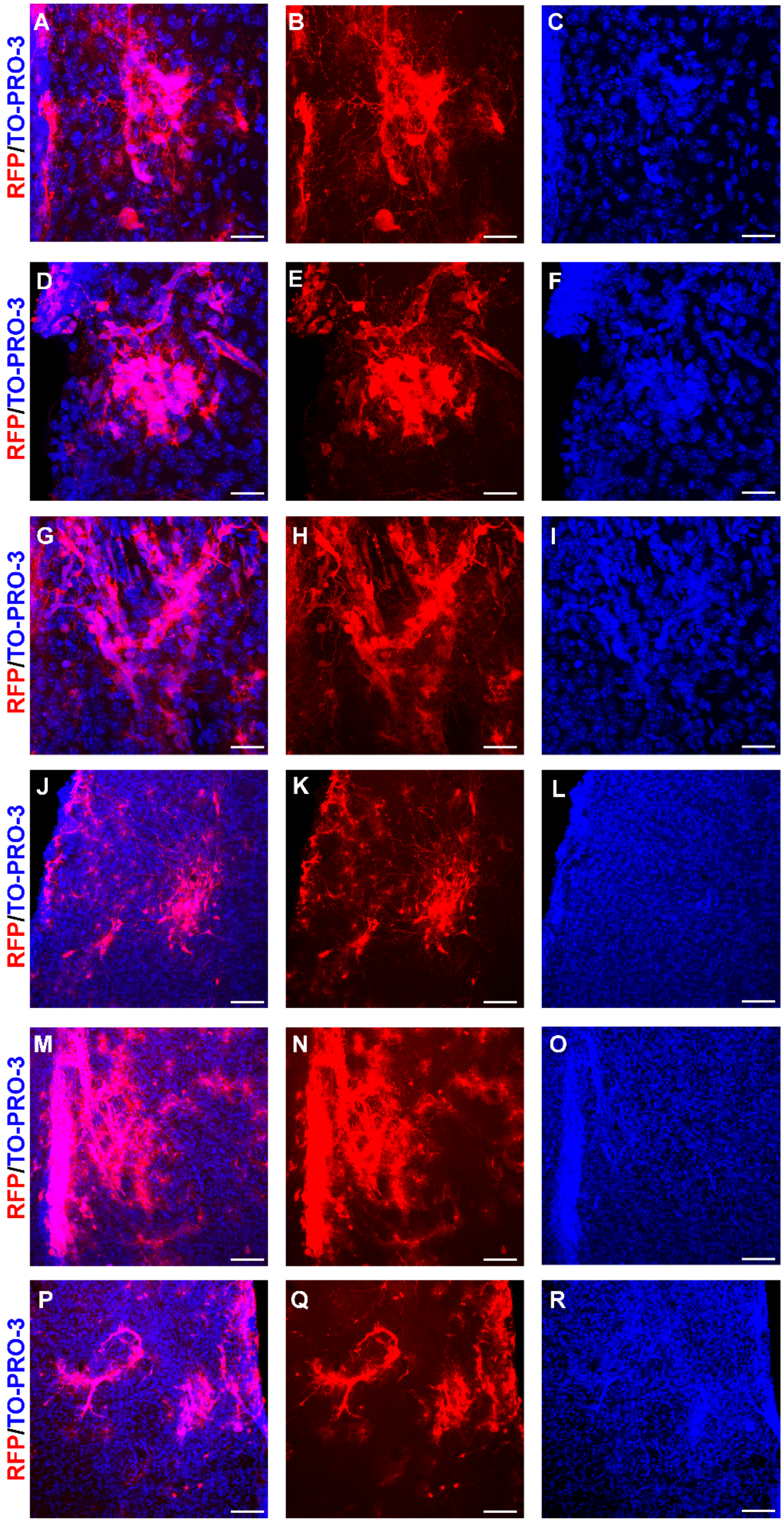

Nuclear counterstains reveal tissue dysplasia in hamartomas of Tsc2 mutant mice, related to Figure 1.
Supplemental Figure 2. A-I) 63x examples of striatal hamartomas showing RFP (red) and nuclei (DNA, TO-PRO-3, blue) counterstaining which demonstrates general disorganization around lesions. Scale bar = 23.8 μm. J-R) 20x examples of striatal hamartomas showing RFP (red) and nuclei (DNA, TO-PRO-3, blue) counterstaining which demonstrates general disorganization around lesions. A-I scale bar = 23.8 μm. J-R scale bar = 75 μm.

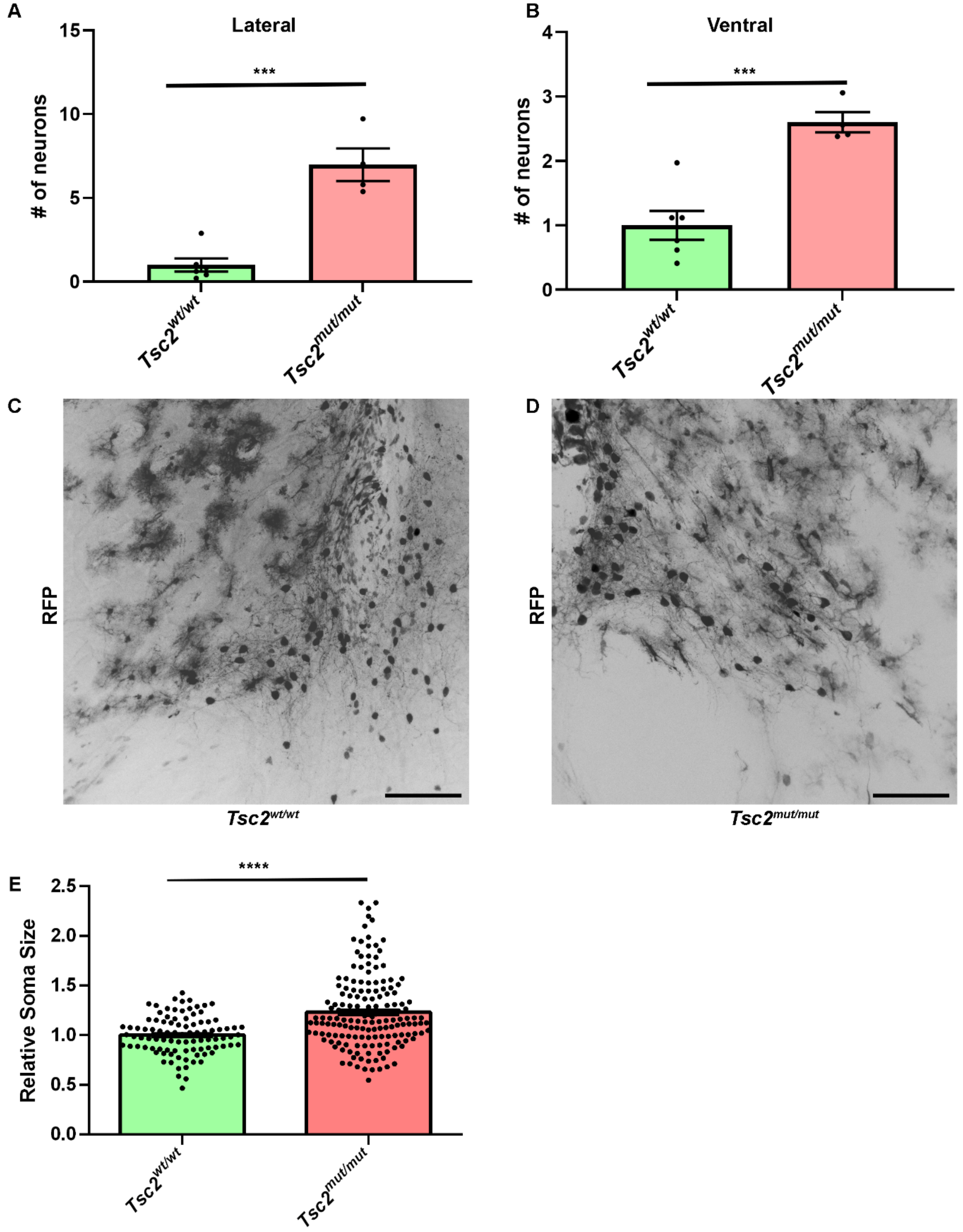

Tsc2 knockout from V-SVZ NSCs generates cytomegalic striatal neurons, related to Figure 1O-R.

Supplemental Figure 3.

A) Quantification of neurons in lateral striatum. $Tsc2^{wt/wt}$, N=6, n=29, mean = 1.000 ± 0.3977 SEM vs. $Tsc2^{mut/mut}$, N=4, n=135, mean = 6.983 ± 0.9791 SEM B) Quantification of neurons in ventral striatum. $Tsc2^{wt/wt}$, N=6, n=204, mean = 1.000 ± 0.2249 SEM vs. $Tsc2^{mut/mut}$, N=4, n=354, mean = 2.603 ± 0.1568 SEM C) Image of $Tsc2^{wt/wt}$ cells in the ventral striatum near the nucleus accumbens. D) Image of $Tsc2^{mut/mut}$ cells in the ventral striatum near the nucleus accumbens. *=*p<0.05*, ***=*p<0.001* E) Quantification of relative soma size. $Tsc2^{wt/wt}$, N=6, n=95, mean

= 1.000 ± 0.0199 SEM vs. *Tsc2$^{mut/mut}$*, N=12, n=164, mean = 1.231 ± 0.0286 SEM. Data are represented as mean ± SEM. Scale bar = 75 µm.

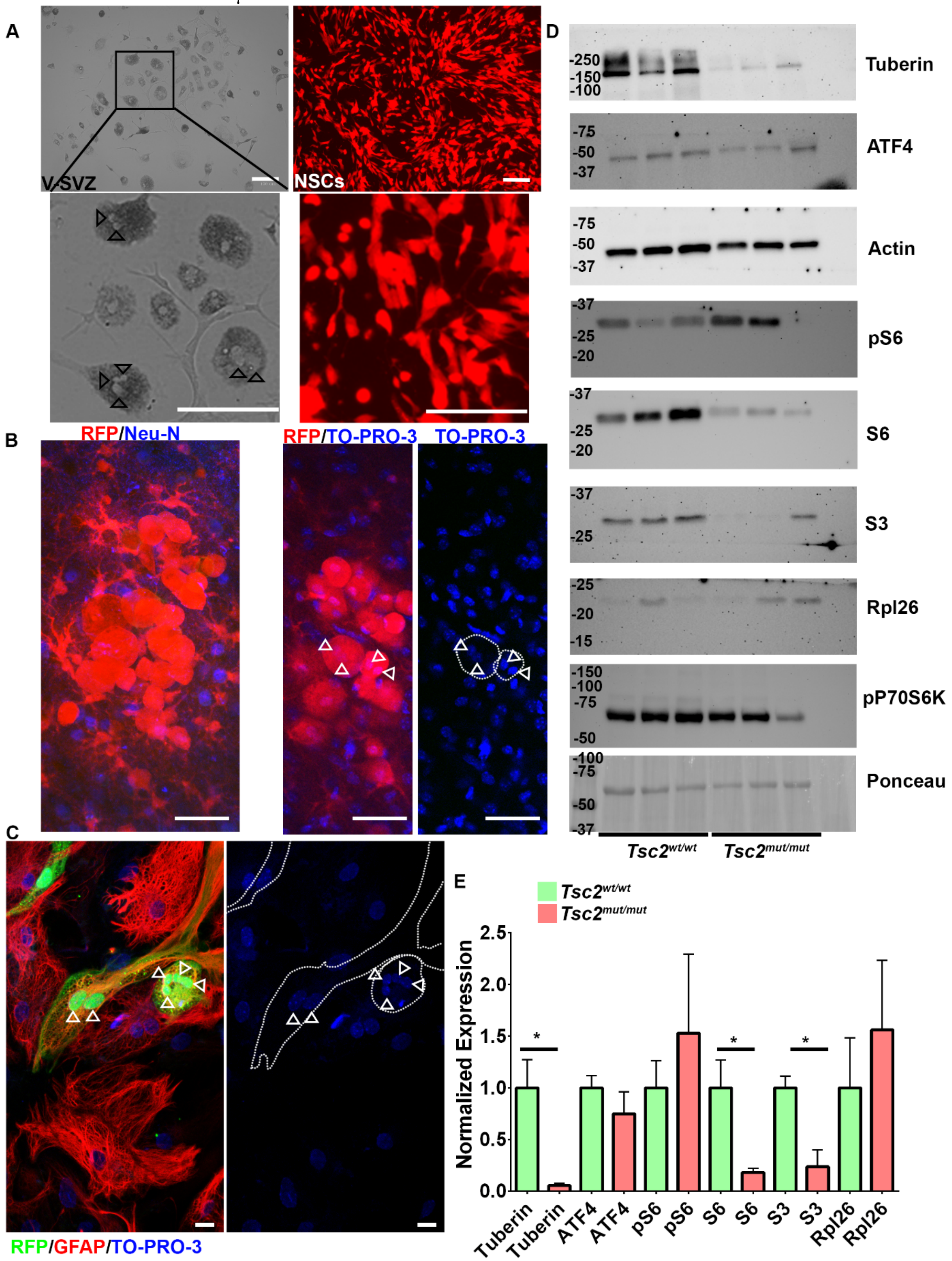

Tsc2 null V-SVZ NSCs generate multinucleated giant cells and have altered translation regulatory proteins, related to Figure 2.

Supplemental Figure 4. A) Left-20x and digitally zoomed transparent image of V-SVZ cells after initial plating. Note the multinucleated giant cells indicated by black arrowheads. Right-20x RFP and digitally zoomed image

demonstrating homogenous morphology after subculturing. B) Left-20x Z-projected confocal image of *Tsc2*$^{mut/mut}$ cells (RFP positive) in the cerebral cortex stained by Neu-N (blue). Center- Section was counterstained with TO-PRO-3 (blue) to visualize individual nuclei. Right- TO-PRO-3 alone with cell body highlighted and arrowheads pointing to nuclei. C) Left-Merged 20x Z-projected confocal image of *Tsc2*$^{mut/mut}$ cells (pseudo-colored Green) from the cortex that are cultured and stained for GFAP (Red) and TO-PRO-3 (Blue) that marks nuclei. Right- TO-PRO-3 alone with cell body highlighted and arrowheads pointing to nuclei. D) Western blots of indicated proteins including tuberin from *Tsc2*$^{wt/wt}$ and *Tsc2*$^{mut/mut}$ NSCs subjected to RNA-sequencing in Figure 2. E) Quantification of western blots in D. *=$p<0.05$ Data are represented as mean ± SEM. A scale bar = 100 μm. B-C scale bar = 23.8 μm.

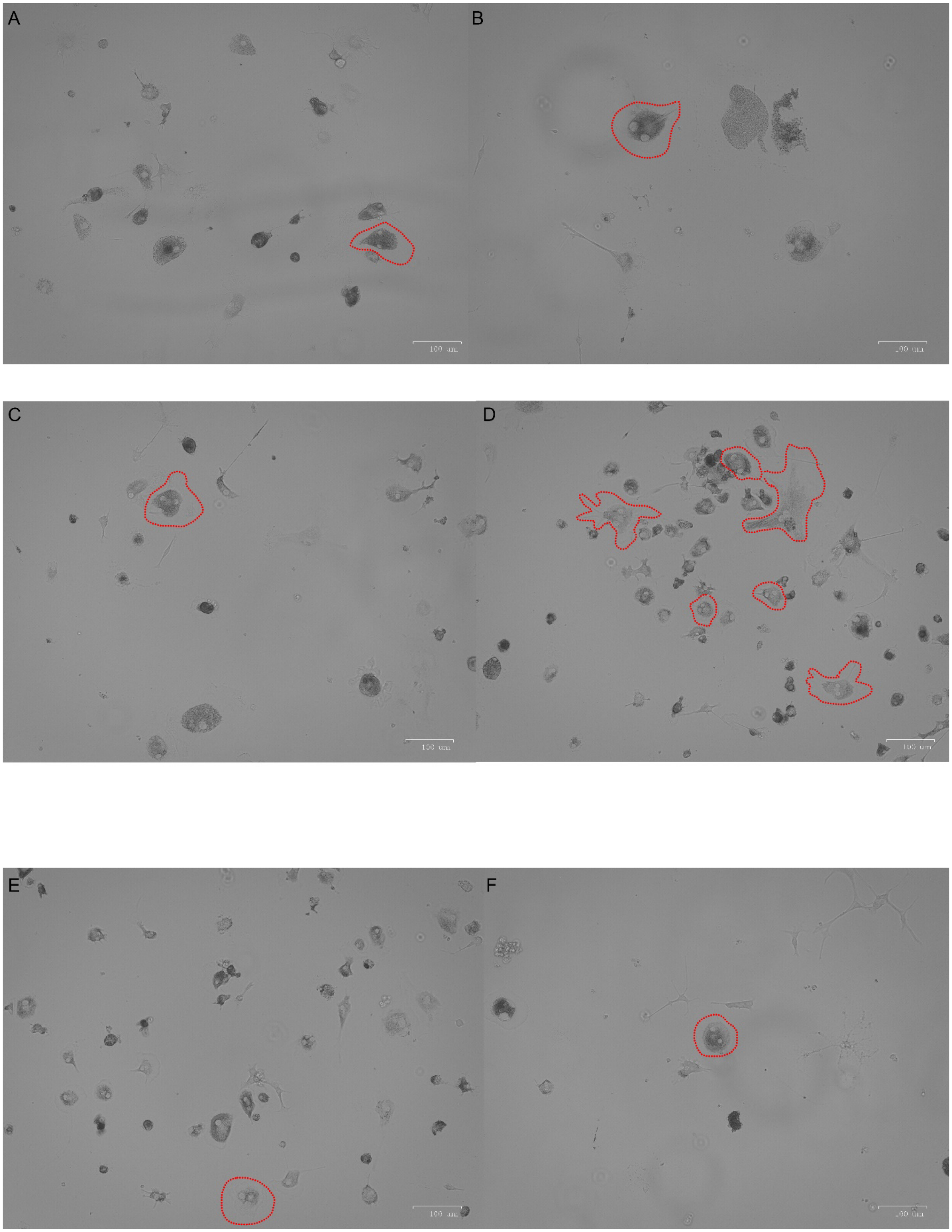

Cultured V-SVZ Cells of Tsc2 Mutant Mice, related to Figure 2.
Supplemental Figure 5. A-F) 20x micrographs of live cells from cultured *V-SVZs* of *Tsc2* x *nestin-CRE-ERT2* neonatal mice injected with tamoxifen and sacrificed at P10. The red dotted line outlines multinucleated cells. Scale bar = 100 μm

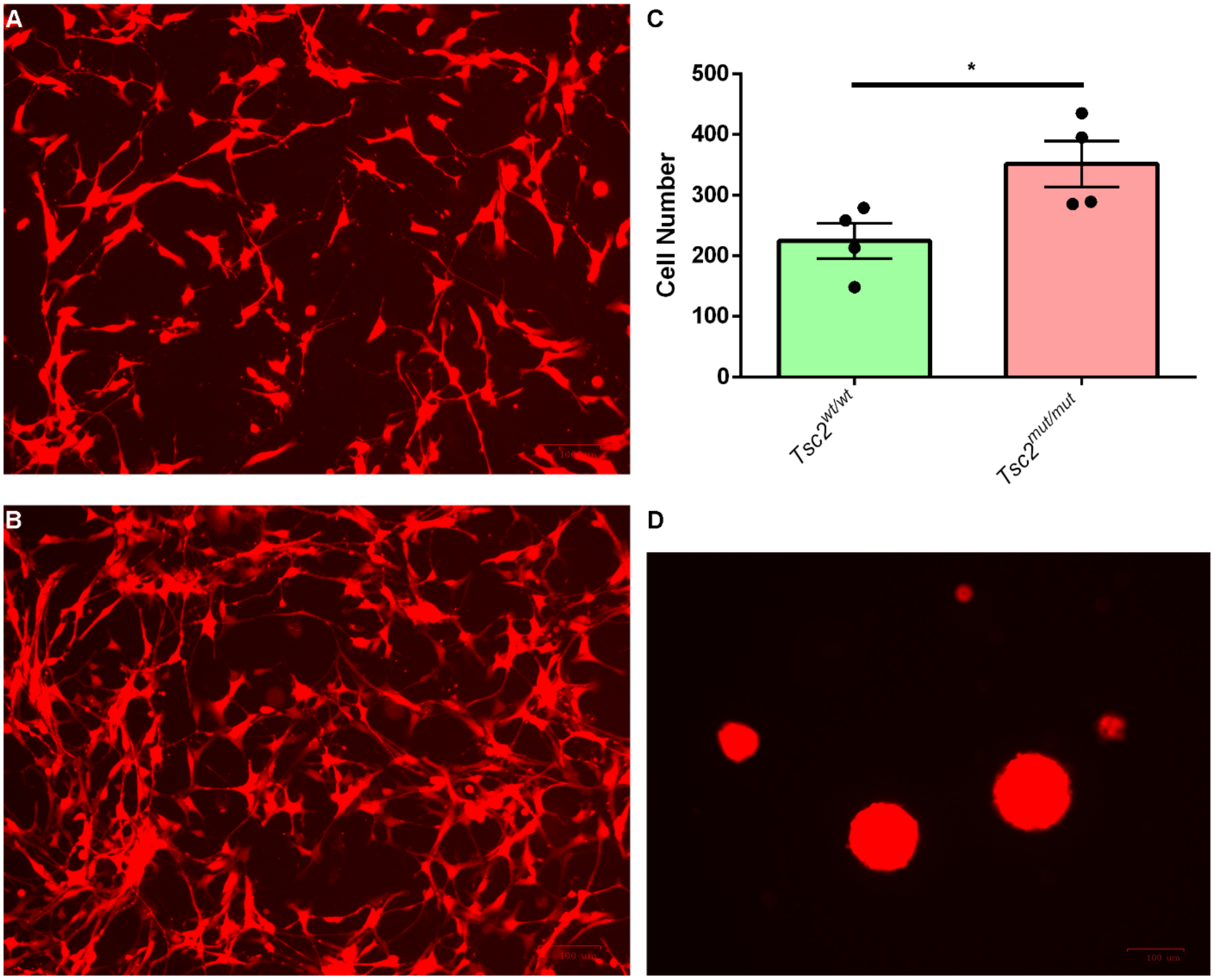

Monolayer NSC Cultures, related to Figure 2.

Supplemental Figure 6. A) 20x image of live *Tsc2*$^{wt/wt}$ NSC culture. B) 20x image of live *Tsc2*$^{mut/mut}$ NSC culture. C) Quantification of cells in long-term NSC cultures. D) Image demonstrating cells can be grown as neurospheres. Data are represented as mean ± SEM.*=$p<0.05$ Scale bar = 100 µm.

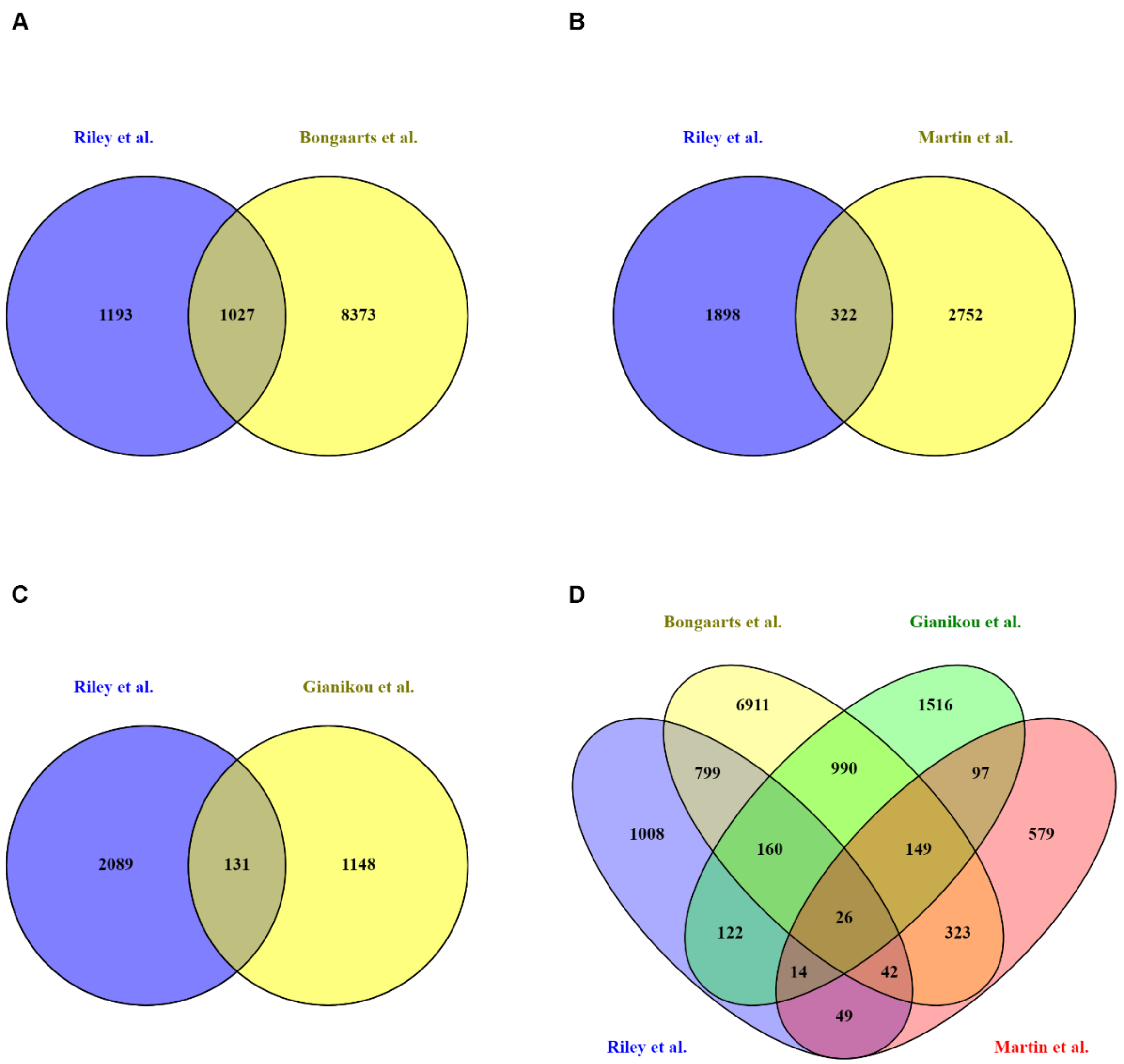

Transcriptome Overlap with Differentially Expressed Transcripts in SEGAs from Bulk RNA Sequencing Studies, related to Figure 2A-B.

Supplemental Figure 7. A) Quantification of overlapping transcripts differentially expressed in NSC cultures from Figure 2 with differentially expressed transcripts identified in SEGA studies[57,75,76]. Note that controls for SEGA studies by Giannikou were in comparison to low grade gliomas.

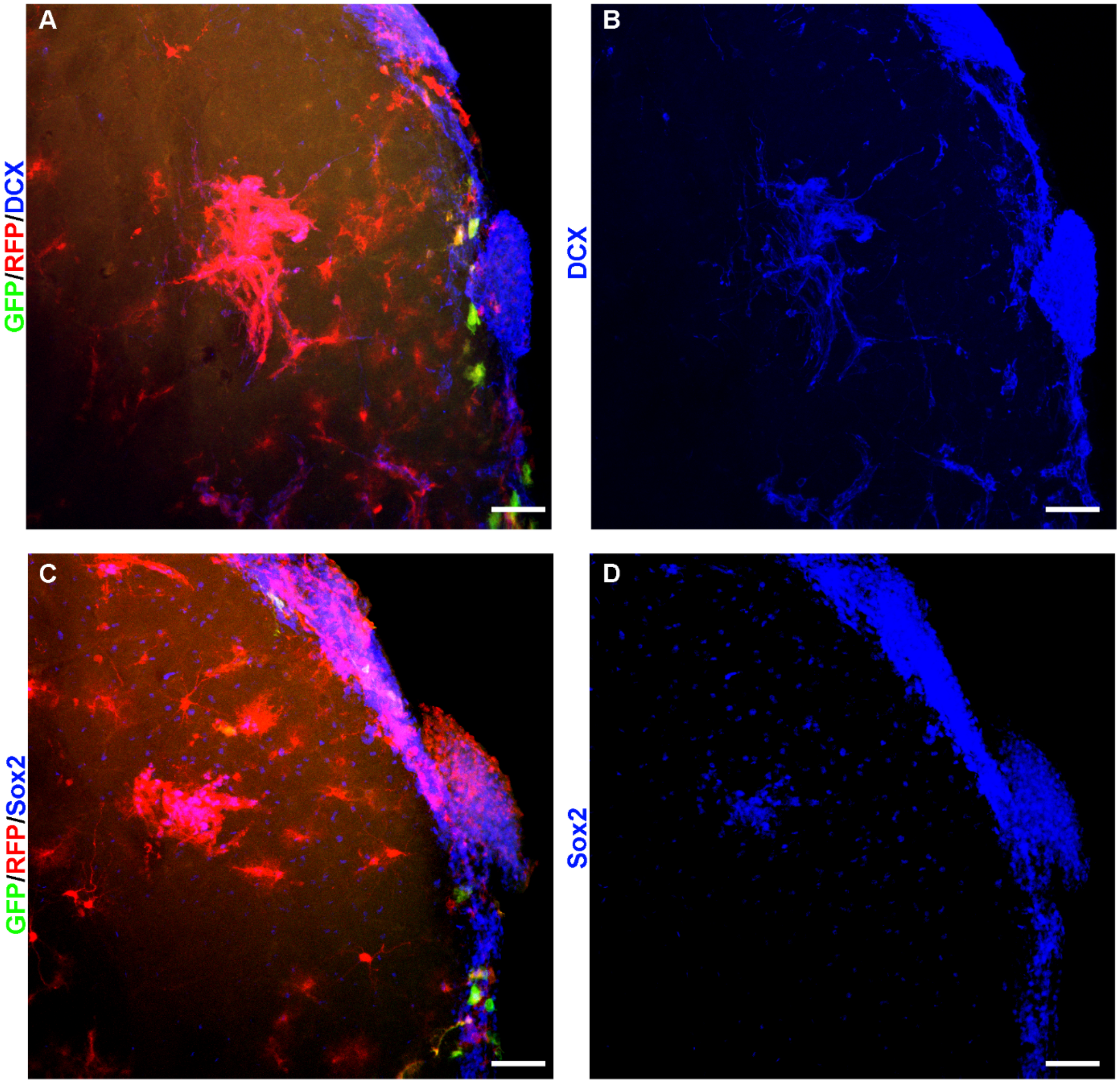

Striatal Hamartoma Staining for DCX and Sox2, related to Figure 4E, F.

Supplemental Figure 8. A-D) Full size 20X images of CRE and GFP electroporated brains with *Tsc2*$^{mut/mut}$ RFP (red) and DCX (A-B, blue) or Sox2 (C-D, blue) in stained hamartomas and nodules as shown in Figure 4. Scale bar = 75 µm.

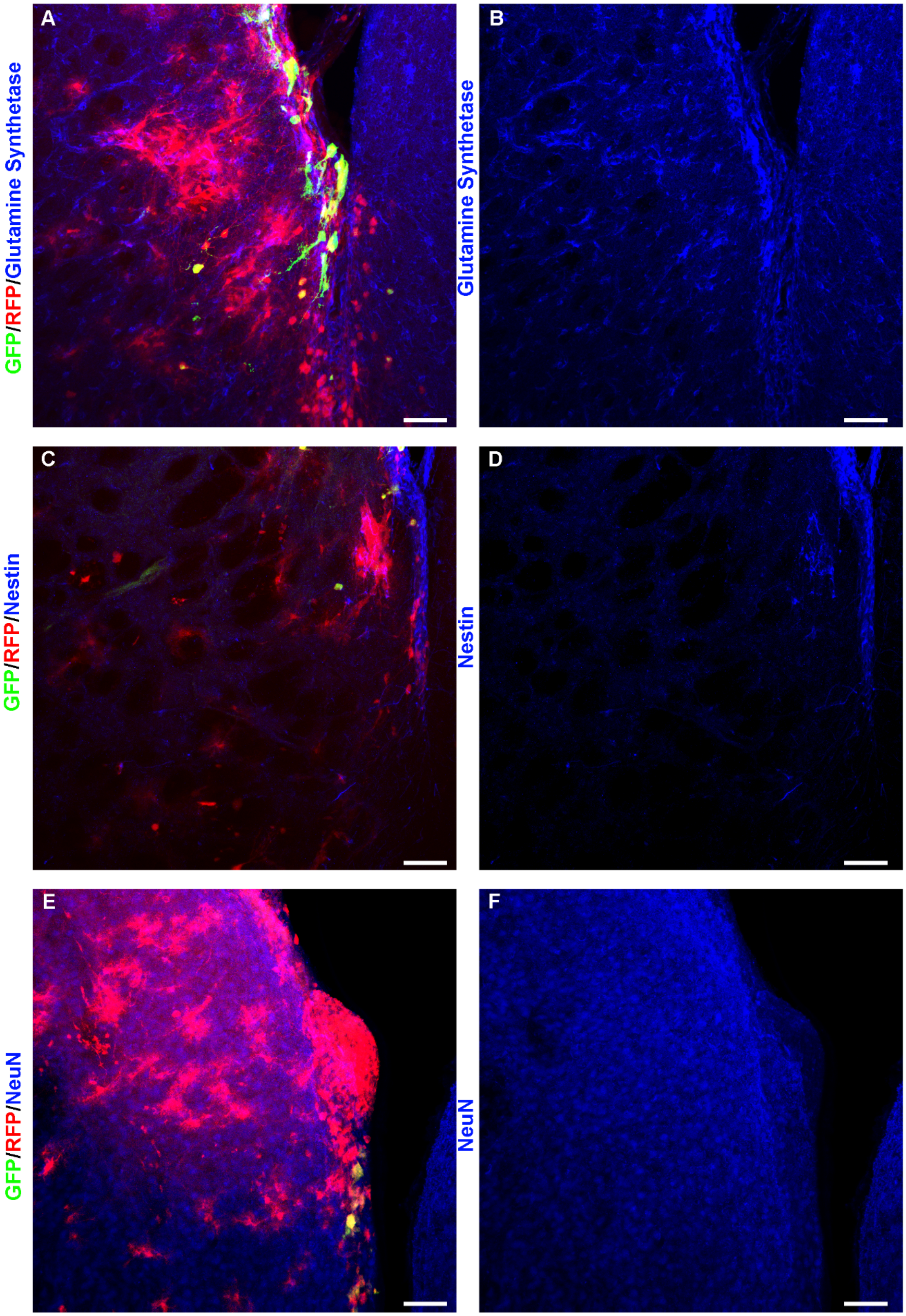

Striatal Hamartoma Staining for Glutamine Synthetase, Nestin, and NeuN, related to Figure 4G-I.
Supplemental Figure 9. A-F) Full size 20x images of Glutamine Synthetase (A-B, blue), Nestin (C-D, blue), or NeuN (E-F, blue) in stained hamartomas and nodules as shown in Figure 4. Scale bar = 75 μm.
